# Efficient Photocatalytic Degradation of Organic Dyes by AgNPs/TiO_2_/Ti_3_C_2_T_*x*_ MXene Composites under UV and Solar Light

**DOI:** 10.1021/acsomega.1c03189

**Published:** 2021-12-01

**Authors:** Zakarya Othman, Alessandro Sinopoli, Hamish R. Mackey, Khaled A. Mahmoud

**Affiliations:** † Qatar Environment and Energy Research Institute (QEERI), Hamad Bin Khalifa University, Qatar Foundation, P.O. Box 34110, Doha, Qatar; ‡ Division of Sustainable Development, College of Science and Engineering, Hamad bin Khalifa University, Qatar Foundation, P.O. Box 34110, Doha, Qatar

## Abstract

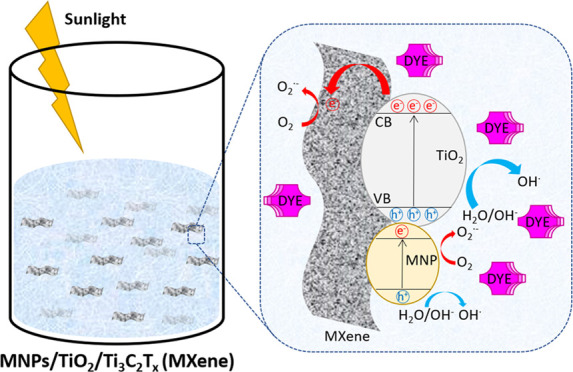

Due to their broad applications in various industrial activities, and their well-known negative impacts on the aquatic environment, organic dyes have been continuously identified as serious threat to the quality of ecosystems. The photocatalytic degradation process in aqueous solutions has emerged as an efficient and reliable approach for the removal of organic dyes. MXenes, a new class of two-dimensional (2D) nanomaterials, possess unique chemical composition, surface functionalities, and physicochemical properties. Such characteristics enable MXenes to act as efficient catalysts or cocatalysts to photodegrade organic molecules. This work explores the application of Ti_3_C_2_T_*x*_ MXene decorated with silver and palladium nanoparticles, using a simple hydrothermal treatment method, for the photocatalytic degradation of methylene blue (MB) and rhodamine B (RhB). The chemical composition of these photocatalysts, as well as their structural properties and morphology, was characterized by scanning electron microscopy (SEM), transmission electron microscopy (TEM), X-ray diffraction (XRD), and X-ray photoelectron spectroscopy (XPS) techniques. The photocatalytic degradation abilities of the pristine MXene and the synthesized MXene composites were investigated under ultraviolet and solar light irradiation. A significant improvement in the photocatalytic performances was observed for all oxidized MXene composites when compared to pristine MXene, with a superior degradation efficiency achieved for AgNPs/TiO_2_/Ti_3_C_2_T_*x*_. This work broadens the application range of oxidized MXene composites, providing an alternative material for degrading organics dyes and wastewater treatment applications.

## 1 Introduction

Synthetic dyes are being used extensively in various industries such as textile, paper, tannery, food, and cosmetic industries. These dyes comprise a principal component of the organic content of effluents produced from such industries. Due to their chemical stability, as well as their toxic and carcinogenic nature, they represent a serious hazard to the recipient environment.^1^ Various advanced water treatment processes have been successfully utilized to remediate dyes from wastewater streams. Among them, photocatalytic degradation has consistently been reported as an effective, low-cost, and green process.^2,3^ Many semiconductor nanomaterials, such as TiO_2_, ZnO, and CdS, have shown high potential for degrading organic contaminants in wastewater.^4−6^ In particular, TiO_2_ is widely reported as one of the best photocatalysts due to its wide band gap, strong oxidizing power, nontoxicity, large surface area, as well as good chemical and photostability.^7,8^ The current research trends consist in developing novel photoactive nanomaterials with improved photocatalytic degradation efficiency by combining two or more materials with tailored optoelectronic properties.

Several TiO_2_-based nanocomposites, such as ZnO/TiO_2_, Fe_2_O_3_/TiO_2_, and organic and nonorganic carbon-based nanomaterials/TiO_2_, have been explored for the degradation of organic pollutants.^9^ In particular, TiO_2_-based nanocomposites with carbon-based materials (e.g., MXene, graphene, and carbon nanotubes) have shown improved photoactivity compared with TiO_2_ alone.^10^ Among the carbon-based nanomaterials, MXene, a novel family of two-dimensional (2D) transition-metal carbides, nitrides, or carbonitrides, has been the subject of several research efforts due to its unique properties including high conductivity, high structural/chemical stability, and abundant hydrophilic functional groups (such as −OH, −O, and −F) on its surface.^11−14^ These advantages make MXene an attractive platform for preparing composites in photocatalytic systems.^13^ In particular, titanium carbide (Ti_3_C_2_T_*x*_) contains a large proportion of Ti, which can undergo surface oxidation to yield TiO_2_/Ti_3_C_2_T_*x*_.^15−18^ For instance, Shahzad et al. fabricated an anatase TiO_2_/Ti_3_C_2_T_*x*_ heterostructure through the hydrothermal treatment process, demonstrating an excellent photocatalytic degradation of the antiepileptic drug carbamazepine.^15^ More importantly, the interfacial Schottky junction that is formed between the TiO_2_ and the layered C atoms provides a large reservoir of holes, which facilitates the charge separation and transfer, essential for the formation of radicals involved in the photodegradation process.^15^

To further enhance the activity of the photocatalysts, the deposition of noble metals on MXene sheets has been reported to improve the photocatalytic activity of the system, attributed to the surface plasmon resonance (SPR) effect and the Schottky barrier formed at the metal–semiconductor interface.^19^ In particular, silver (Ag) has attracted large attention due to its high catalytic activity, high electrical conductivity, and relatively lower cost compared to other noble metals.^20^ For example, Ag metal doping of ZnO/graphene photocatalysts has been proven to be an efficient route for achieving higher degradation efficiency of methyl orange because of the effective charge separation therein.^21^ Additionally, Ag nanoparticles can be easily deposited on the surface by self-reduction of silver salts (e.g., AgNO_3_), where MXene acts as the redox agent, leading to nucleation and growth of spherical Ag nanoparticles on the surface of MXene nanosheets.^22^ Nevertheless, few works in the literature studied the incorporation of silver onto MXene sheets for catalytic applications.^20,22−24^ For example, Huang et al. prepared AgNP-loaded MXene/Fe_3_O_4_/polymer nanocomposites through a self-reduction reaction process, which resulted in excellent catalytic degradation and cycle stability toward nitroaromatic compounds.^22^ Together with Ag, palladium (Pd) represents another example of noble metals recently used in designing catalysts with improved photodegradation activity.^25,26^ Pd is one of the most active elements for interacting with the surface of various oxides and exhibits remarkable catalytic properties.^27^ For instance, MXene/polymer nanocomposites decorated with Pd nanoparticles, through the self-reduction of Pd^2+^ ions by MXene, have improved the catalytic activity of the nanocomposites and resulted in excellent catalytic reduction performance of nitro compounds, such as 2-nitrophenol and 4-nitrophenol.^26^

Herein, and in light of the potential effects of Ag, Pd, and TiO_2_ on the enhancement of the photocatalytic activity of MXene, we demonstrate a simple one-pot hydrothermal deposition of Ag (or Pd) and in situ hydrothermal growth of TiO_2_ on Ti_3_C_2_T_*x*_ sheets, from a solution containing delaminated (DL) Ti_3_C_2_T_*x*_ nanosheets and Ag (or Pd) metal salt. Ti_3_C_2_T_*x*_, TiO_2_/Ti_3_C_2_T_*x*_, AgNPs/TiO_2_/Ti_3_C_2_T_*x*_, and PdNPs/TiO_2_/Ti_3_C_2_T_*x*_ composites have been prepared and characterized. The photocatalytic activity of the four composites has been investigated and compared. The selection of these competing composites is intended to evaluate the impact of the oxidation process and the influence of different noble metals’ deposition on the photocatalytic performance and properties of the Ti_3_C_2_T_*x*_. All composites were studied under both ultraviolet (UV) and solar light for the degradation of methylene blue (MB) and rhodamine B (RhB) as precursor organic dye pollutants and widely used as a benchmark for photocatalytic activity of the novel photocatalysts. The most performing photocatalyst has been successfully adopted on a real wastewater sample by monitoring the variation of the total organic carbon (TOC).

## 2 Results and Discussion

### 2.1 Morphological Investigations

**Figure 1:**
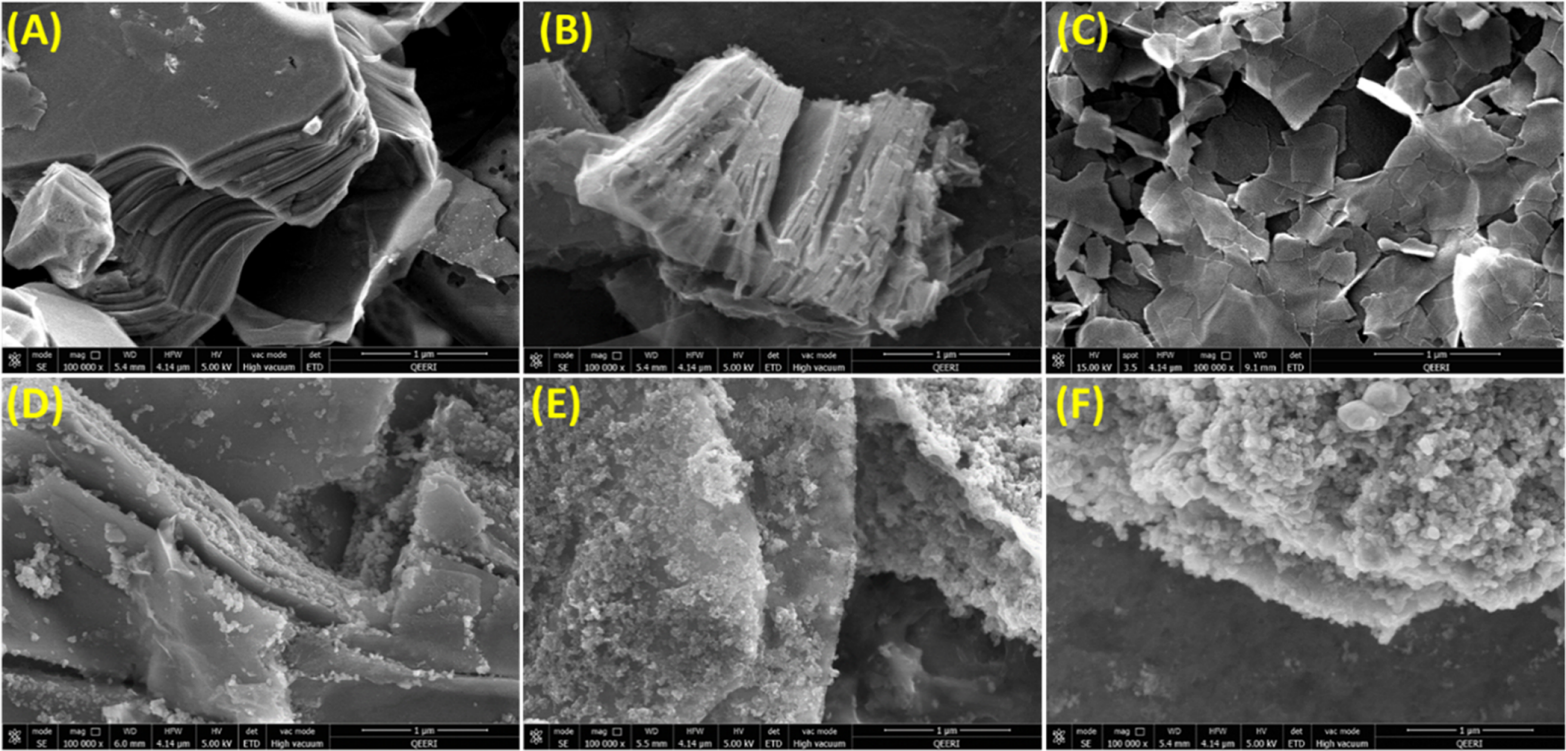
SEM images of (A) Ti_3_AlC_2_ MAX phase, (B) ML-Ti_3_C_2_T*_x_* MXene, (C) DL-Ti_3_C_2_T_*x*_ MXene, (D) TiO_2_/Ti_3_C_2_T_*x*_, (E) PdNPs/TiO_2_/Ti_3_C_2_T_*x*_, and (F) AgNPs/TiO_2_/Ti_3_C_2_T_*x*_ photocatalysts.

The morphology and microstructure of the Ti_3_AlC_2_ MAX phase, multilayered (ML)-Ti_3_C_2_T*_x_* MXene, DL-Ti_3_C_2_T*_x_* MXene, TiO_2_/Ti_3_C_2_T*_x_*, PdNPs/TiO_2_/Ti_3_C_2_T*_x_*, and AgNPs/TiO_2_/Ti_3_C_2_T*_x_* composites were investigated by scanning electron microscopy (SEM). The Ti_3_AlC_2_ MAX phase has a compact layered structure (Figure 1A). The accordion-like multilayer structure confirms the typical MXene morphology (Figure 1B), which was then delaminated to single- or few-layered MXene through in situ ultrasonication (Figure 1C). After thermal treatment, TiO_2_ nanoparticles were formed on MXene sheets and increased the surface roughness of the material (Figure 1D). The resulting morphologies confirm the successful seeding and growth of Ag and Pd nanoparticles onto the oxidized-MXene surface (Figure 1E,F), together with a further enhancement of the surface roughness.

**Figure 2:**
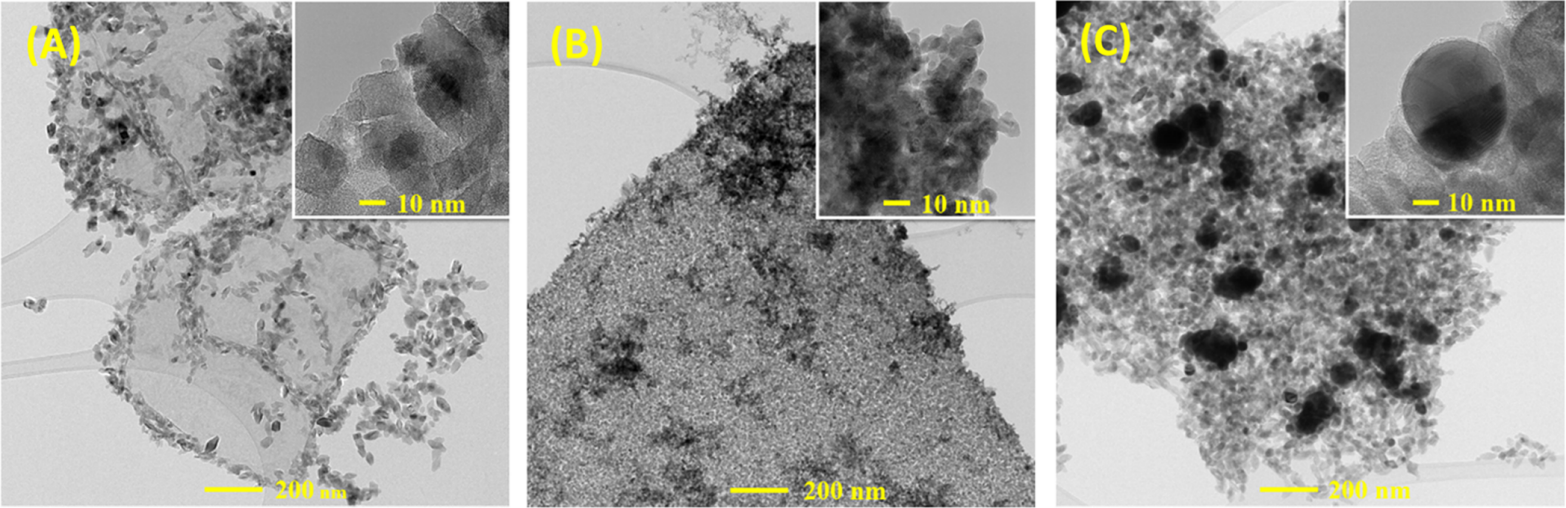
TEM images of (A) TiO_2_/Ti_3_C_2_T_*x*_, (B) PdNPs/TiO_2_/Ti_3_C_2_T_*x*_, and (C) AgNPs/TiO_2_/Ti_3_C_2_T_*x*_.

To further investigate the microstructure of the samples, transmission electron microscopy (TEM) analysis was performed. While Figure S1 shows individual sheets of MXene, Figure 2A shows the formation of TiO_2_ nanocrystals on MXene sheets, starting from the sheet edges and continuing until covering the entire material surface.^28^ For the PdNPs/TiO_2_/Ti_3_C_2_T_*x*_ photocatalysts, a mixture of nanoparticles and nanoagglomerates of palladium was formed (Figure 2B). The formation of agglomerated Pd particles was expected as it has been previously reported.^29^Figure 2C displays the AgNPs/TiO_2_/Ti_3_C_2_T_*x*_ photocatalysts, where various shapes and size distributions of AgNPs were formed besides the TiO_2_ onto the surface of MXene sheets. The diameter of individual silver particles ranges between 30 and 100 nm. The distributions of O, Ti, Pd, and Ag within the flakes were investigated with high-angle annular dark-field scanning transmission electron microscopy (HAADF-STEM) for all samples, and the corresponding element maps are shown in Figures S2–S5. The images further confirmed the presence of the desired nanoparticles and showed that they were homogeneously dispersed throughout the whole composites.

**Figure 3:**
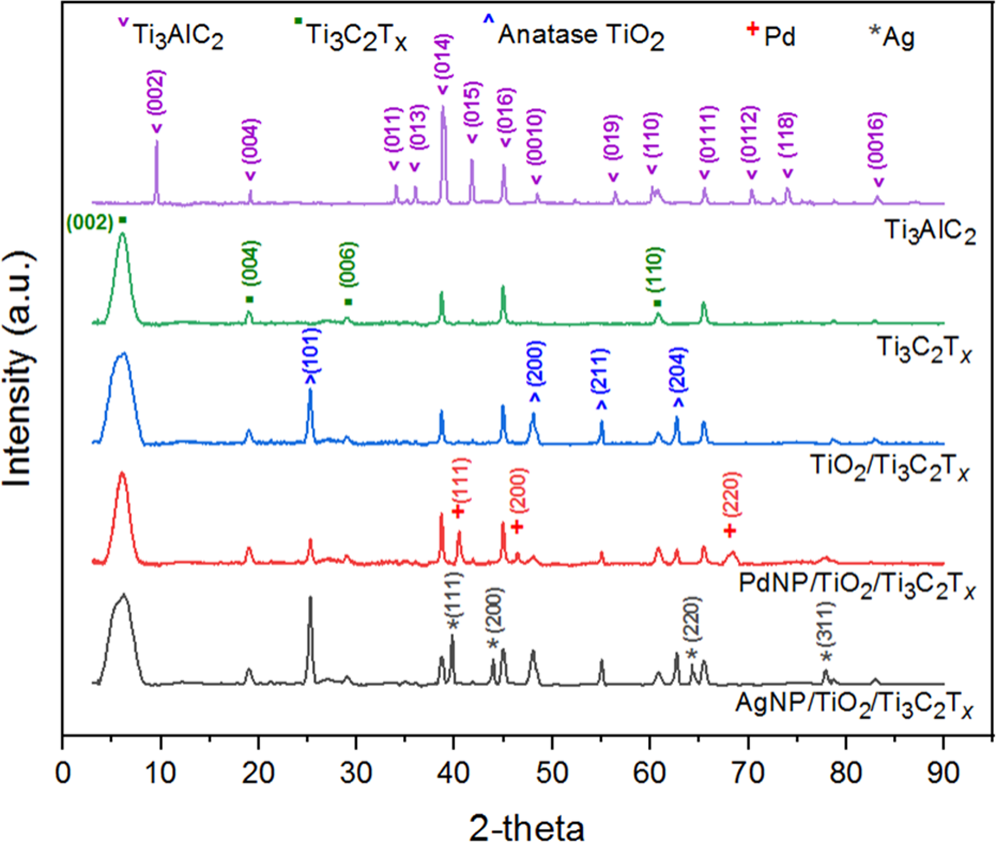
XRD patterns for Ti_3_AlC_2_ MAX phase, DL-Ti_3_C_2_T*_x_* MXene, TiO_2_/T_3_C_2_T*_x_*, PdNPs/TiO_2_/T_3_C_2_T*_x_*, and AgNPs/TiO_2_/T_3_C_2_T*_x_* photocatalysts. In parentheses are the indices for the corresponding planes.

X-ray diffraction (XRD) analysis was obtained to confirm the crystalline phases of the materials. As shown in Figure 3, the diminishing of the characteristic MAX peaks, such as at 2θ = 34.03, 36.02, 38.80, 41.82, and 45.04°, indicates the successful etching of the Ti_3_AlC_2_ MAX phase and formation of exfoliated MXene.^30,31^ Additionally, the shifting and broadening of the (002) characteristic MAX peak to the lower angle (2θ = 6.82°) indicate the accommodation of surface terminations and intercalants within MXene interlayer spacing.^31^ Furthermore, all of the XRD patterns show the characteristic peaks of Ti_3_C_2_T*_x_* at 2θ = 6.82, 18.86, 28.79, and 60.61°, which are, respectively, assigned to the (002), (004), (006), and (110) planes, similar to previous reports.^20,30−33^ The XRD pattern of TiO_2_/Ti_3_C_2_T*_x_* shows additional characteristic diffraction peaks at 25.23, 48.02, 55.01, and 62.63°, corresponding to anatase TiO_2_ (JCPDS no. 21-1272).^34^ Thus, the presence of anatase TiO_2_ nanoparticles in XRD patterns is attributed to the partial oxidation of MXene sheets, using an indigenous buildup of anatase TiO_2_ particles from the titania layers of MXene during the hydrothermal oxidation. The XRD pattern of AgNPs/TiO_2_/Ti_3_C_2_T*_x_* showed another four additional pronounced diffraction peaks at 2θ values of 39.74, 43.92, 64.28, and 77.92° characteristic of the (111), (200), (220), and (311) planes of the Ag single crystal (JCPDS no. 04-0783),^1,35^ confirming successful reduction of Ag nanoparticles on the MXene surface. Similarly, for PdNPs/TiO_2_/Ti_3_C_2_T*_x_* nanocomposites, the characteristic diffraction peaks for Pd nanoparticles were also observed.^36^

**Figure 4:**
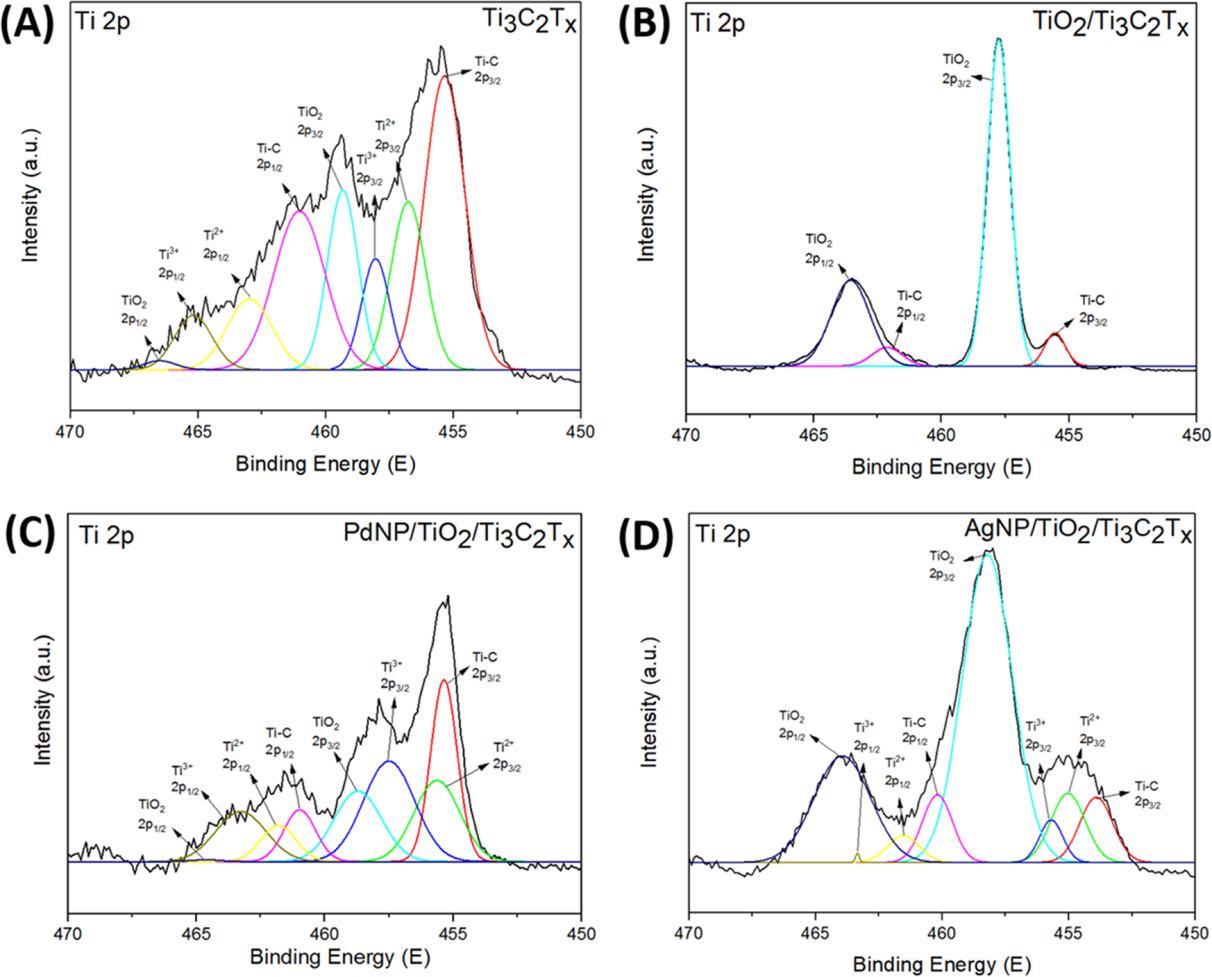
Ti 2p XPS spectra for (A) Ti_3_C_2_T_*x*_, (B) TiO_2_/Ti_3_C_2_T_*x*_, (C) PdNPs/TiO_2_/Ti_3_C_2_T_*x*_, and (D) AgNPs/TiO_2_/Ti_3_C_2_T_*x*_ photocatalysts.

**Figure 5:**
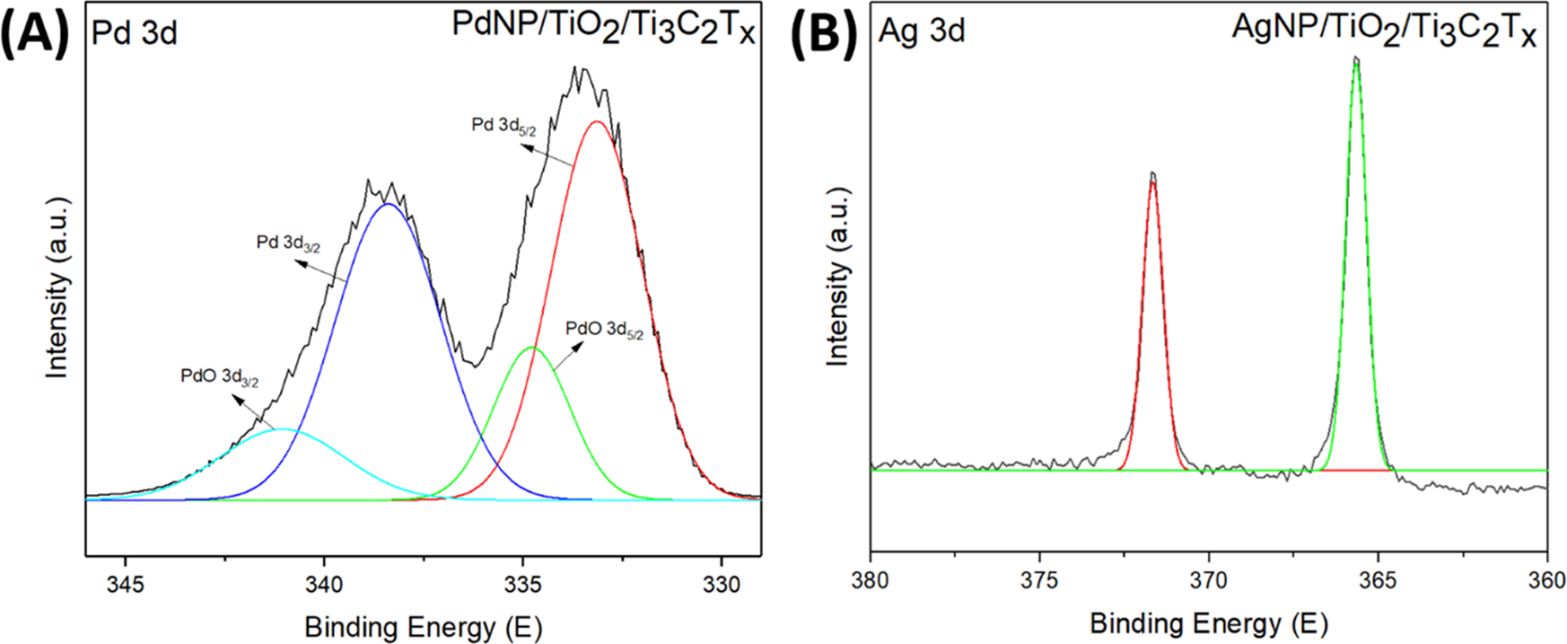
XPS spectra for (A) Pd 3d for PdNPs/TiO_2_/Ti_3_C_2_T_*x*_, and (B) Ag 3d for AgNPs/TiO_2_/Ti_3_C_2_T_*x*_ photocatalysts.

X-ray photoelectron spectroscopy (XPS) was carried out to examine the surface elemental composition and chemical states of pristine MXene and corresponding composites. As described in Table S1, a significant increase in oxygen content was noticed for the oxidized MXene composites (TiO_2_/Ti_3_C_2_T_*x*_, AgNPs/TiO_2_/Ti_3_C_2_T_*x*_, and PdNPs/TiO_2_/Ti_3_C_2_T_*x*_), which confirms the oxidation of the titanium layer to TiO_2_ nanocrystals on the MXene surface. Fluorine is present as a consequence of the use of lithium fluoride during the MAX phase etching process and was lower in all oxidized MXene composites in comparison with the pristine Ti_3_C_2_T_*x*_, which could be attributed to the hydrothermal treatment effect and the following centrifugal washing steps. The deconvoluted peaks of Ti_3_C_2_T_*x*_, TiO_2_/Ti_3_C_2_T_*x*_, AgNPs/TiO_2_/Ti_3_C_2_T_*x*_, and PdNPs/TiO_2_/Ti_3_C_2_T_*x*_ samples are shown in Figure S6, except for Ti 2p (Figure 4), Pd 3d, and Ag 3d (Figure 5).

The Ti 2p spectra of Ti_3_C_2_T_*x*_ showed two doublets, which were deconvoluted into eight peaks at 466.6, 465.0, 462.5, 461.2, 458.9, 457.7, 455.7, and 454.4 eV. These peaks belong to TiO_2_ 2p_1/2_, Ti^3+^ 2p_1/2_, Ti^2+^ 2p_1/2_, Ti–C 2p_1/2_, TiO_2_ 2p_3/2_, Ti^3+^ 2p_3/2_, Ti^2+^ 2p_3/2_, and Ti–C 2p_3/2_, respectively (Figure 4A). These results are consistent with the previous reports.^37−40^ The fixed area ratio of Ti 2p_3/2_ and Ti 2p_1/2_ is around 2:1 with a doublet separation of 5.7 eV. By comparing the valence of Ti in the pristine Ti_3_C_2_T_*x*_ nanosheets with the prepared TiO_2_/Ti_3_C_2_T_*x*_ nanocomposite, we can notice the disappearing of the area fraction of Ti(I), Ti(II), and Ti(III) species and an increase in the area fraction of Ti(IV), indicating a transformation of Ti from Ti(I), Ti(II), and Ti(III) to Ti(IV). This confirms the formation of TiO_2_ through the hydrothermal process (Figure 4B). For the PdNPs/TiO_2_/Ti_3_C_2_T_*x*_, the addition of Pd salts has limited the thermal treatment oxidation effect on MXene, resulting in quite a similar Ti 2p spectrum to that of pristine Ti_2_C_3_T*_x_* (Figure 4C). On the contrary, for AgNPs/TiO_2_/Ti_3_C_2_T_*x*_, the addition of silver salts has a significant influence on promoting the oxidation of MXene up to almost similar levels of TiO_2_/Ti_3_C_2_T_*x*_ (Figure 4D).

Figure 5 shows the Pd 3d and Ag 3d XPS spectra of PdNPs/TiO_2_/Ti_3_C_2_T_*x*_ and AgNPs/TiO_2_/Ti_3_C_2_T_*x*_, respectively. For the PdNPs/TiO_2_/Ti_3_C_2_T_*x*_, the Pd ions were either reduced to metallic Pd nanoparticles or oxidized to pallidium oxides. As shown in Figure 5A, the XPS spectrum of Pd 3d has doublet peaks that could be deconvoluted to four peaks, two main peaks centered at 333.3 and 338.4 eV matching with Pd 3d_5/2_ and Pd 3d_3/2_ of metallic Pd, and two weak peaks centered at 334.9 and 341.4 eV matching with Pd(II) 3d_5/2_ and Pd(II) 3d_3/2_ of palladium oxide (PdO).^41^ However, for AgNPs/TiO_2_/Ti_3_C_2_T_*x*_, the Ag ions were reduced to metallic Ag without producing silver oxides, which could boost the oxidation of MXene and the formation of TiO_2_ crystals. The Ag 3d XPS spectrum of AgNPs/TiO_2_/Ti_3_C_2_T_*x*_ showed distinct peaks at 365.7 and 371.7 eV, which were assigned to Ag 3d_5/2_ and Ag 3d_3/2_ peaks, respectively, indicating the successful self-reduction of Ag nanoparticles on the MXene surface (Figure 5B).^19,42^

The major peaks fitted in the Ti_3_C_2_T_*x*_ C 1s profile were at 281.9, 284.9, 286.8, and 288.7 eV corresponding to C–T, C–C, C–O, and COO bonds of Ti_3_C_2_T_*x*_. The C 1s high-resolution XPS fitting profile for AgNPs/TiO_2_/Ti_3_C_2_T*_x_* showed a significant drop in the intensity of the C–T bond energy together with a slight shift in the binding energy compared to the spectrum of Ti_3_C_2_T_*x*_, ascribed to the formation of TiO_2_ nanoparticles at the expense of oxidation of C–T bonds of Ti_3_C_2_T_*x*_ sheets (Figure S7).^43^ For O 1s spectra (Figure S8), the peaks at 529.5, 531.3, and 532.0 eV were attributed to Ti–O–Ti, Ti–O, and terminal C–O bonds of Ti_3_C_2_T_*x*_. After hydrothermal treatment, the Ti–O–Ti peak increased significantly with a notable shift of other peaks to the low binding energies, indicating distinctive dominance of TiO_2_ on TiO_2_/Ti_3_C_2_T_*x*_ and AgNPs/TiO_2_/Ti_3_C_2_T_*x*_ surfaces.^43^

The charge separation efficiency of the photocatalysts was investigated via photoluminescence (PL) spectroscopy. The efficiency of photogenerated electron–hole pairs is generally inversely proportional to the relative intensity of the PL spectrum.^11^ As shown in Figure S11, the obtained spectra confirm the improved charge separation efficiency of the synthesized photocatalysts. The inclusion of metal nanoparticles (MNPs) (Ag or Pd) results in substantial quenching of the TiO_2_/Ti_3_C_2_T_*x*_ PL intensity, which results in AgNPs/TiO_2_/T_3_C_2_T_*x*_ and PdNPs/TiO_2_/T_3_C_2_T_*x*_ photocatalysts displaying the fastest and second fastest charge separation, respectively. This indicates that the inclusion of metal nanoparticles can effectively limit the rapid charge recombination of photogenerated charge carriers, hence increasing the catalytic performances.

### 2.2 Photocatalytic Degradation Studies

#### 2.2.1 Effect of Catalyst-to-Dye Ratio under UV Light

**Figure 6:**
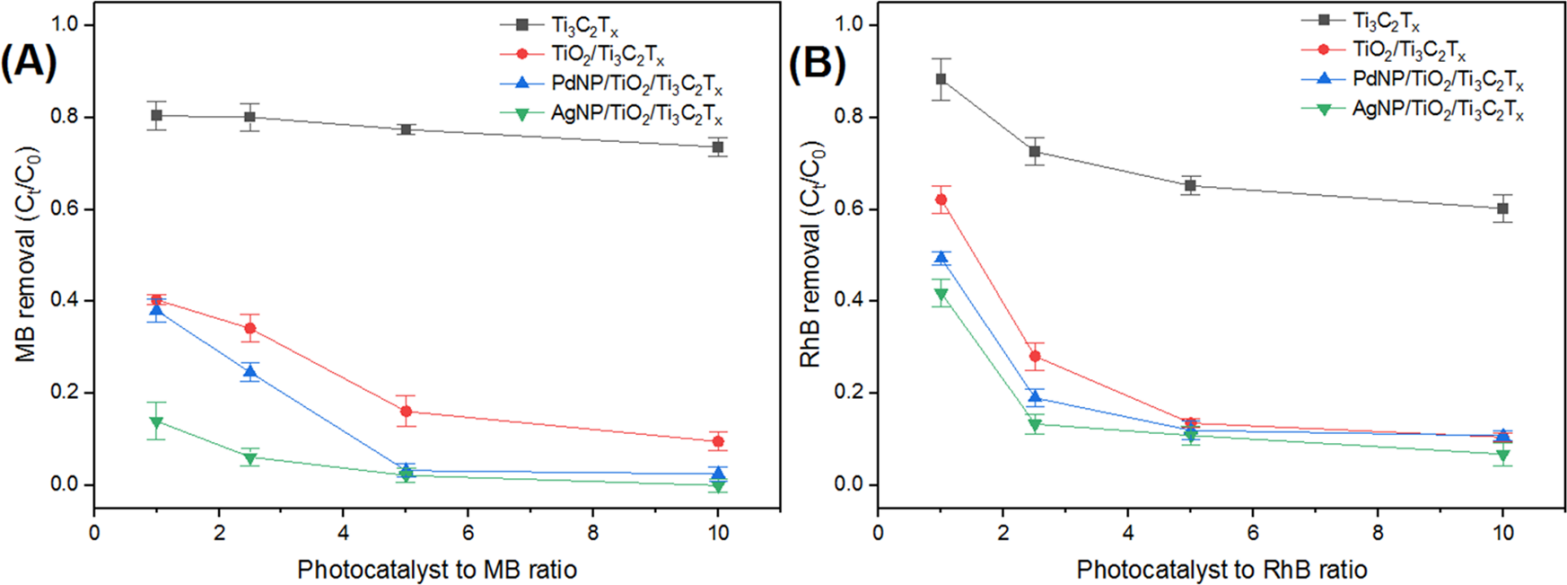
Effect of photocatalyst-to-dye ratios on degradation performance under UV light for 15 min of (A) MB and (B) RhB.

The as-prepared composites MXenes were tested for the photocatalytic degradation of MB and RhB dyes under UV light irradiation for 15 min. The effect of the photocatalyst-to-dye ratio was examined by varying the photocatalyst amount from 1 to 10 mg against a fixed concentration of MB or RhB (10 mg/L) in 100 mL of deionized (DI) water. As shown in Figure 6, the three composite MXene photocatalysts showed rapid and superior degradation rates, against both MB and RhB, compared to pristine Ti_3_C_2_T_*x*_ MXene at all studied ratios. In particular, AgNPs/TiO_2_/Ti_3_C_2_T_*x*_ showed up to 85% MB degradation at a low photocatalyst-to-dye ratio (1:1). The percentage increased at higher ratios and reached full degradation at the ratio 10:1 within 15 min only. Additionally, AgNPs/TiO_2_/Ti_3_C_2_T_*x*_ also exhibits the optimal performance for the degradation of RhB at all dosages as compared with PdNPs/TiO_2_/Ti_3_C_2_T_*x*_ and TiO_2_/Ti_3_C_2_T_*x*_. The improved photocatalytic performance of these MXene nanocomposites could be mainly ascribed to the formation of anatase TiO_2_ particles on the surface MXene substrates. It was also noticed that the presence of noble metals led to a slightly better performance, which could be attributed to the surface plasmonic resonance effect, and hence to their role in enhancing charge separation.^44^ It is expected that yielding more TiO_2_ nanocrystals, as observed from XPS analysis, would boost the photocatalytic activity of AgNPs/TiO_2_/Ti_3_C_2_T_*x*_ in comparison to PdNPs/TiO_2_/Ti_3_C_2_T_*x*_ photocatalysts.^15,45^ Moreover, the slightly faster kinetics of AgNPs/TiO_2_/Ti_3_C_2_T*_x_* in comparison to PdNPs/TiO_2_/Ti_3_C_2_T_*x*_ could also be attributed to the size and distribution of the nanoparticles. While the silver nanoparticles have a large semispherical structure, the palladium particles formed nanoagglomerates (Figure 2B,C), which limit their activity.^26,29^ Additionally, as seen in the XPS spectrum for Pd 3d (Figure 5A), a reasonable amount of palladium was present as Pd^2+^ rather than Pd^0^. This could result in an undesired shift of the absorption band edge and limit the SPR effect of Pd^0^ metal nanoparticles, hence affecting the catalytic activity.^46^

#### 2.2.2 Effect of Catalyst-to-Dye Ratio under Solar Light

**Figure 7:**
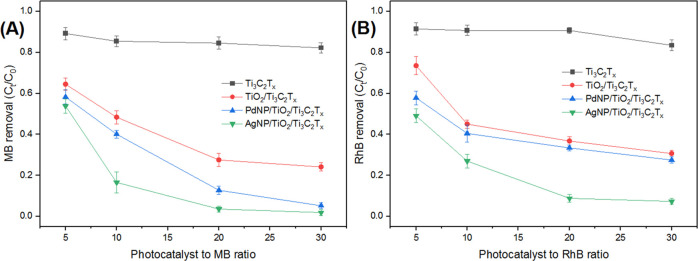
Effect of photocatalyst-to-dye ratios on degradation performance under solar light for 120 min of (A) MB dye and (B) RhB.

Photocatalytic performance of MXene composites was also examined under solar light irradiation over 120 min. As shown in Figure S9, the largest component of generated light by the AM 1.5 filter is in the visible (vis) region. In all experiments, various photocatalyst-to-dye ratios were investigated to better compare the performances of all prepared photocatalysts. As shown in Figure 7, AgNPs/TiO_2_/Ti_3_C_2_T_*x*_ displayed better performance than PdNPs/TiO_2_/Ti_3_C_2_T_*x*_ with more than 90% removal efficiency of MB and RhB at higher photocatalyst-to-dye ratios (20:1 and 30:1). Results were further confirmed by UV–vis absorption spectroscopy (Figure S10), where the surface plasmon resonance effect for Ag and Pd played a crucial role in enhancing the absorption in the visible range of light and enhancing the charge separation step.^24^ On the other hand, as reported for another titania-based photocatalyst, the anatase TiO_2_ particles also played an important role in harvesting the high-energy UV light component of the solar spectrum, as can be anticipated from the TiO_2_/Ti_3_C_2_T_*x*_ performance. No significant degradation was observed by the pristine Ti_3_C_2_T_*x*_ over 120 min under solar light.

For most of the as-synthesized MXene composites, the dye removal efficiency increased significantly up to certain photocatalyst-to-dye ratios, specifically 2.5–5:1 (under UV light, Figure 6) and 10–20:1 (under solar light, Figure 7). Above such ratios, the dye removal efficiency did not increase significantly. This is due to the shielding effect of 2D materials that can affect the light penetration, which is more pronounced at high catalyst loadings.^47^ On the other hand, owing to the increase in the number of photocatalyst active sites, the degradation efficiency at higher photocatalyst loading ratios was still the highest. For the loading studies, the point where the 2D shielding effect overcomes the effect of a higher number of active sites was not reached.

#### 2.2.3 Effect of UV and Solar Irradiation Time and Photocatalysis Kinetics

The loadings corresponding to the highest removal with the lowest ratio have been selected for the kinetic studies, in particular, 2.5:1 and 20:1 photocatalyst-to-dye ratios were selected for the subsequent experiments under UV light and solar light, respectively. It is worth noting that the required degradation time for the solar light experiments was much longer than that for the UV light experiments, even though the photocatalyst dosage was much higher. In general, this can be attributed to the setup for the UV light experiments, which allows better harvesting of all of the light emitted by the source as compared to the external setup for solar simulator experiments,^48^ and to higher absorption of the synthesized catalysts in the UV rather than in the visible spectral range.

**Figure 8:**
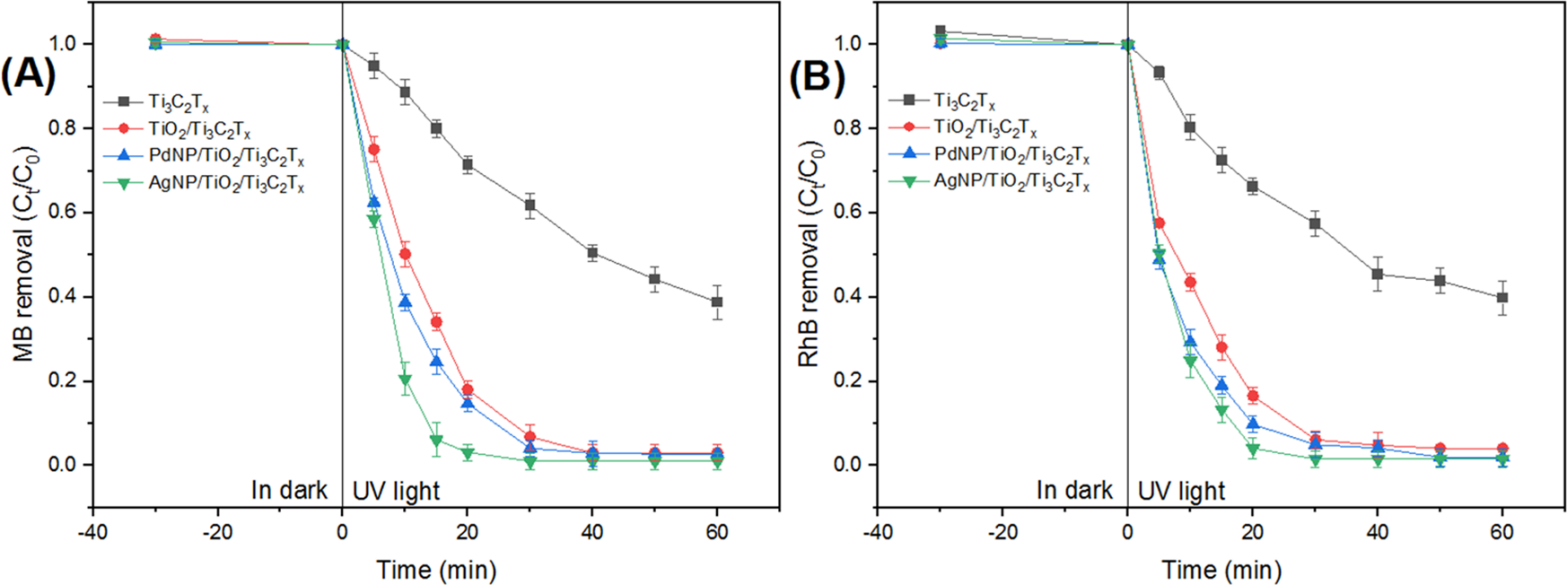
Effect of UV irradiation time on the degradation performance by the synthesized photocatalysts (2.5:1 photocatalyst-to-dye ratio) of the MB dye (A) and RhB (B).

**Figure 9:**
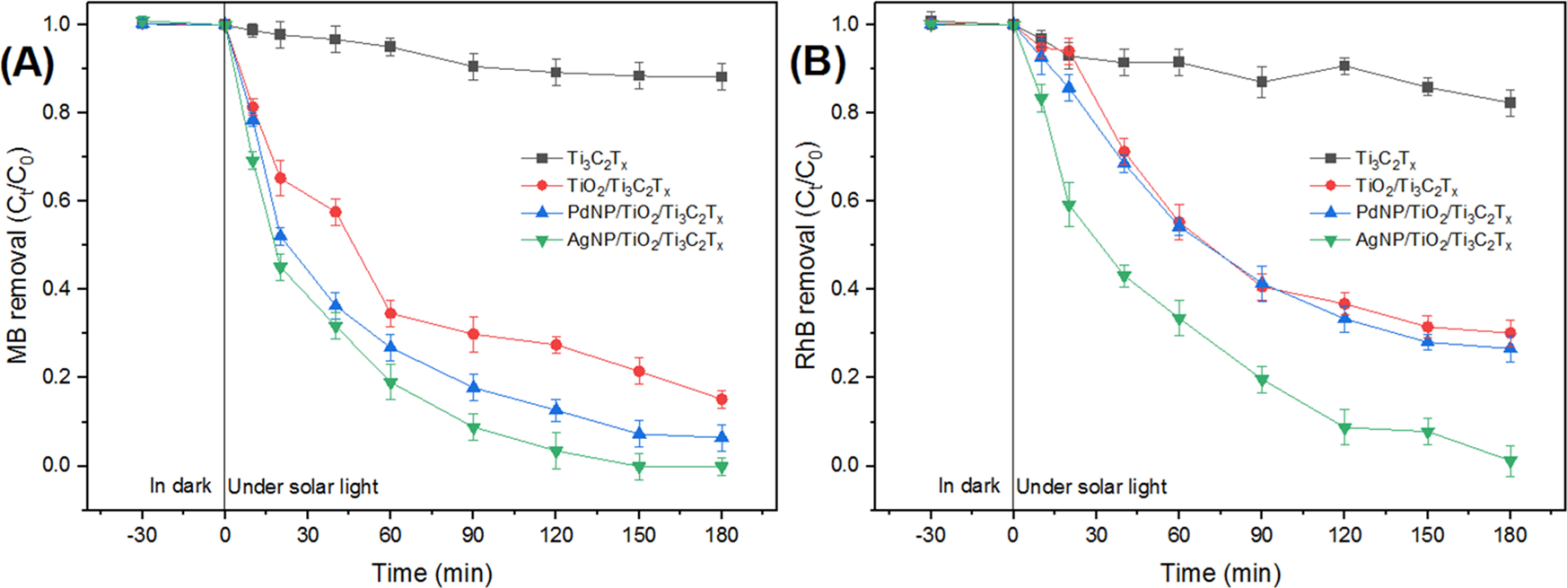
Effect of solar light irradiation time on the degradation performance by the synthesized photocatalysts (20:1 photocatalyst-to-dye ratio) of MB (A) and RhB (B).

Determining the degradation kinetic rates is crucial in practical applications for examining the photocatalytic performances against organic dyes. In this context, experiments were performed with the two optimal ratios of 2.5:1 under a UV light ratio and 20:1 under solar light to investigate the effect of irradiation time on the photocatalytic degradation of MB and RhB. It was found that AgNPs/TiO_2_/Ti_3_C_2_T_*x*_ exhibited the fastest kinetic response and its photocatalytic degradation efficiency against MB and RhB increased rapidly within the initial 15 min under UV light and the first 60 min under solar light, to reach a plateau (Figures 8 and 9). Similar response trends were also observed with PdNPs/TiO_2_/Ti_3_C_2_T_*x*_ and TiO_2_/Ti_3_C_2_T_*x*_. It is reasonable to expect that as the MB or RhB concentration decreases over time, the chances of interaction between photocatalysts and dye molecules decrease too. Further, it was reported that the degradation of such dyes leads to the formation of intermediate subspecies (such as azure A, azure B, azure C, thionin for MB dye and ethanediotic acid, 1,2-benzenedicarboxylic acid, 4-hydroxy benzoic acid, and benzoic acid for RhB dye) that compete themselves with the original dyes for the active sites at the surface of the photocatalyst.^49,50^

To quantify the photocatalytic activity of AgNPs/TiO_2_/Ti_3_C_2_T_*x*_ as the best photocatalyst here, the corresponding photodegradation data for MB and RhB were correlated with the simplified form of the Langmuir–Hinshelwood kinetic model, the pseudo-first-order kinetic model, as follows^51^
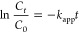
1where *C_t_* and *C*_0_ are the final and initial concentrations of MB or RhB (mg/L), respectively. *k*_app_ is the apparent rate constant (min^–1^), and *t* is the time (min).

**Figure 10:**
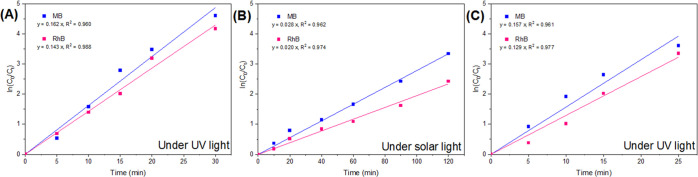
Kinetic trends for photodegradation of MB and RhB by AgNPs/TiO_2_/Ti_3_C_2_T_*x*_ (A) under UV light and (B) under solar light. (C) Kinetic trends for photodegradation in the copresence of MB and RhB by AgNPs/TiO_2_/Ti_3_C_2_T_*x*_.

The apparent rate constant (*k*_app_) and the goodness-of-fit measure *R*_2_ are shown in Figure 10. Results clearly showed that the photocatalytic activities of AgNPs/TiO_2_/Ti_3_C_2_T_*x*_ matched well with the pseudo-first-order kinetics model for both dyes under UV light and solar light irradiation.

The observed photodegradation kinetics of MB was generally faster than that of RhB with the AgNPs/TiO_2_/Ti_3_C_2_T_*x*_ photocatalyst, under the same experimental conditions (Figure 10). These results match well with other works in the literature and can be attributed mainly to the different adsorption energies of MB and RhB toward metal nanoparticles.^52,53^ Specifically, the nitrogen and sulfur atoms of the thiazine core in MB are well-known to interact with metals (in particular silver), and RhB, with its carboxylic and amino groups, has demonstrated capability to adsorb on metal surfaces and carbon materials. Additionally, MXene composites generally possess a high negative charge density in aqueous solutions, which helps in the adsorption of cationic dyes such as MB. RhB dye has both positive and negative charges associated with its structure, and hence electrostatic interactions may not be very strong with MXene composites.^54,55^ Different adsorption affinities of the investigated dyes toward the photocatalyst surface can be easily seen from the variation of the UV–vis spectrum during degradation with AgNPs/TiO_2_/Ti_3_C_2_T_*x*_ in the copresence of MB and RhB dyes (Figure S12). Degradation of MB resulted in a decrease of the corresponding absorption peak (664 nm) faster than that of the RhB peak (555 nm), together with an associated blue shift of the MB peak, indicating the formation of intermediate subspecies. The effect of UV irradiation time on photodegradation of coexisting MB and RhB by AgNPs/TiO_2_/Ti_3_C_2_T_*x*_ is shown in Figure S13, and the photodegradation kinetics followed a pseudo-first-order as indicated in Figure 10C.

#### 2.2.4 Comparison of MB and RhB Degradation by AgNPs/TiO_2_/Ti_3_C_2_T*_x_* with Other Reported Photocatalysts

The degradation efficiency of MB and RhB by the prepared AgNPs/TiO_2_/Ti_3_C_2_T_*x*_ and the pseudo-first-order rate constants were compared with the performance of some MXenes, graphene, activated carbon, and metal oxide composite photocatalysts reported in the literature. As listed in Table 1, AgNPs/TiO_2_/Ti_3_C_2_T_*x*_ resulted in degradation efficiency and kinetic rate constant toward MB and RhB, under UV irradiation, higher than most of the previously reported photocatalysts, including metal-oxide-based photocatalysts. Additionally, under solar irradiation, AgNPs/TiO_2_/Ti_3_C_2_T_*x*_ demonstrated degradation performances comparable to other reported systems such as RGO/BiOI/AgI and graphene/TiO_2_. It is worth mentioning that the listed photodegradation efficiencies are strongly influenced by the experimental condition; therefore, the comparison with our systems is merely speculative. Nevertheless, the reported photocatalytic properties suggested that the synthesized AgNPs/TiO_2_/Ti_3_C_2_T_*x*_ could be an excellent candidate for the photocatalytic degrading of organic dyes in the aqueous media.

**Table 1:** Degradation Performance Comparison of MB and RhB by Various Photocatalysts under UV and Solar Irradiation

catalyst	dye	light source	catalyst/dye ratio	degradation rate/constant	ref
CeO_2_/Ti_3_C	RhB	500 W Hg lamp (UV)	50:1	75% (90 min)	(56)
Ti@Ag_ZnO-SS	RhB	Hg lamp (UV)		95% (120 min)	(57)
Ag-ZnO/g-C_3_N_4_	MB	Hg lamp (UV)	60:1	96% (60 min)	(58)
TiO_2_/AC	MB	15 W lamp (UV)	≈62:1	88% (90 min)	(59)
TiO_2_/rGO	MB	24 W (UV)	50:1	93% (240 min), *K* = 0.052 min^–1^	(60)
Mg-doped ZnO	RhB	125 W Hg lamp (UV)	2.5:1	78% (120 min), *K* = 0.013 min^–1^	(61)
AgNPs/TiO_2_/Ti_3_C_2_T_*x*_	MB RhB	400 W Hg lamp (UV)	2.5:1	99% MB (30 min), *K* = 0.162 min^–1^	this work
				99% RhB (40 min), *K* = 0.143 min^–1^	
Ni/NiO/TiO_2_	RhB, MB	simulated solar light	500:1	74.4% RhB (30 min), 98% MB (30 min)	(62)
BiOBr/Ti_3_C_2_	RhB	300 W xenon lamp (solar light)	10:1	99.4% (24 min)	(63)
TiO_2_-GO	MB	500 W Hg lamp (solar light)	10:1	95% (50 min), *K* = 0.052 min^–1^	(64)
Mg-doped ZnO	MB	100 W xenon lamp (solar light)	50:1	98% (120 min)	(65)
MOF-5@rGO	MB RhB	solar simulator	≈156:1	93% MB (20 min)	(66)
			≈104:1	97% RhB (20 min)	
graphene–TiO_2_	RhB	xenon lamp (solar light)	≈42:1	98% (60 min)	(67)
rGO/BiOI/AgI	RhB	150 W Xe lamp (solar light)	100:1	83% (90 min), *K* = 0.018 min^–1^	(68)
AgNPs/TiO_2_/Ti_3_C_2_T_*x*_	MB RhB	simulated solar light	20:1	96% MB (120 min), *K* = 0.028 min^–1^	this work
				88% RhB (120 min), *K* = 0.020 min^–1^	

#### 2.2.5 Application of AgNPs/TiO_2_/Ti_3_C_2_T*_x_* to Real Wastewater

**Figure 11:**
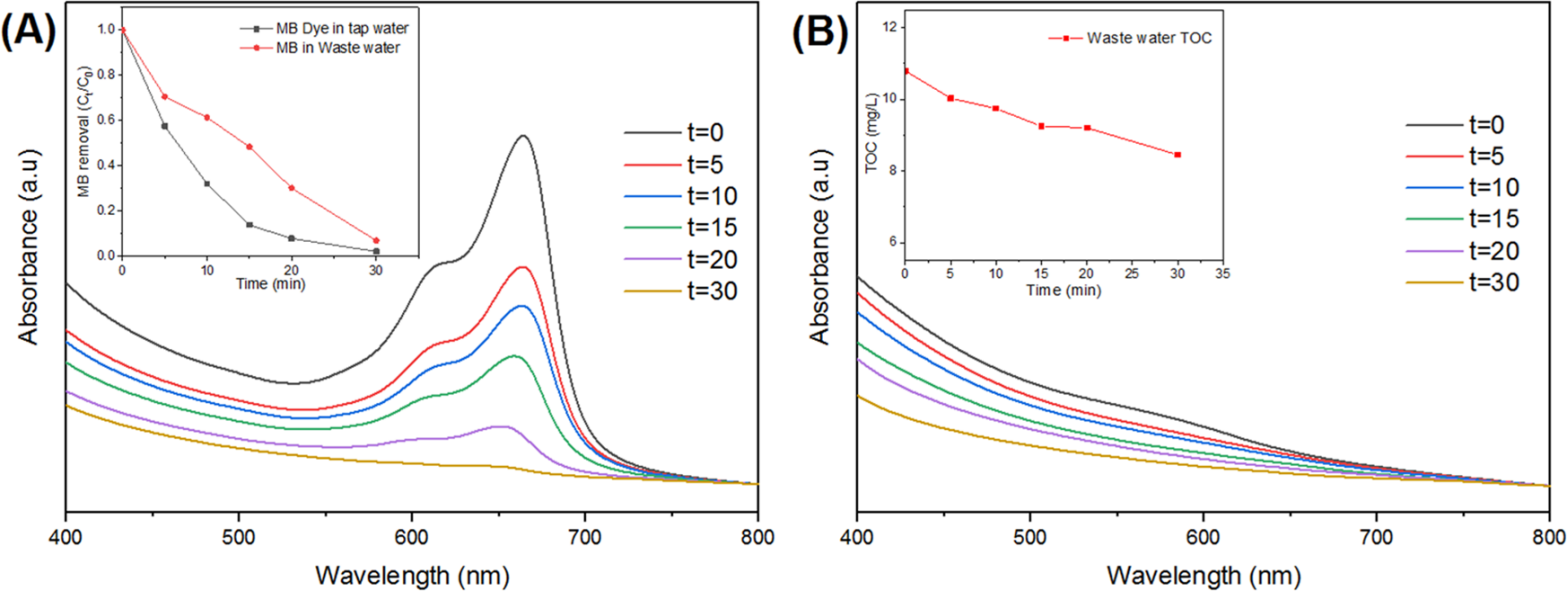
(A) UV–visible spectrum of photocatalytic degradation of AgNPs/TiO_2_/Ti_3_C_2_T_*x*_ against wastewater spiked with MB. Inset: comparison of MB removal from tap water and wastewater under UV light irradiation by AgNPs/TiO_2_/Ti_3_C_2_T_*x*_. (B) UV–visible spectrum of photocatalytic degradation of AgNPs/TiO_2_/Ti_3_C_2_T_*x*_ against wastewater. Inset: total organic carbon removal from wastewater under UV light irradiation by AgNPs/TiO_2_/Ti_3_C_2_T_*x*_.

To assess the practical dye degradation application of AgNPs/TiO_2_/Ti_3_C_2_T_*x*_, tap water wastewater samples were collected from a local paper recycling factory and were tested under UV irradiation. For a better comparison with the previous photocatalytic degradation experiments conducted in this study, both samples were spiked with MB dye to obtain a final MB concentration of 10 mg/L. Figure 11A demonstrates the MB removal from wastewater and tap water, under the same operational conditions, with a 2.5:1 photocatalyst-to-MB dye ratio. From the inset, the MB degradation response in wastewater was slightly slower than that in tap water, which can be attributed to the presence of other competing organics in the wastewater. Nevertheless, it could be clearly seen that almost complete removal of MB occurred. The high MB removal efficiencies from various water bodies demonstrate the great application potential of AgNPs/TiO_2_/Ti_3_C_2_T_*x*_ in removing organic dyes from wastewater.

The photocatalytic degradation of unspiked wastewater by AgNPs/TiO_2_/Ti_3_C_2_T_*x*_ was also investigated. While the absorption profile of wastewater does not show any strong band, the measured UV–vis spectra clearly show that the entire spectrum is dimming, suggesting a decrease in the overall concentration of organic species, which was further confirmed by the decrease in the total organic carbon. The total organic carbon was reduced by 23% over 30 min of irradiation (inset, Figure 11B). The lower rate of removal of native wastewater TOC, in comparison to MB, is speculatively attributed to the different affinities of the various organic species present with the photocatalyst, in comparison to MB, which instead has a strong affinity toward the AgNPs/TiO_2_/Ti_3_C_2_T_*x*_ photocatalyst.

### 2.3 Proposed Photodegradation Mechanism

**Figure 12:**
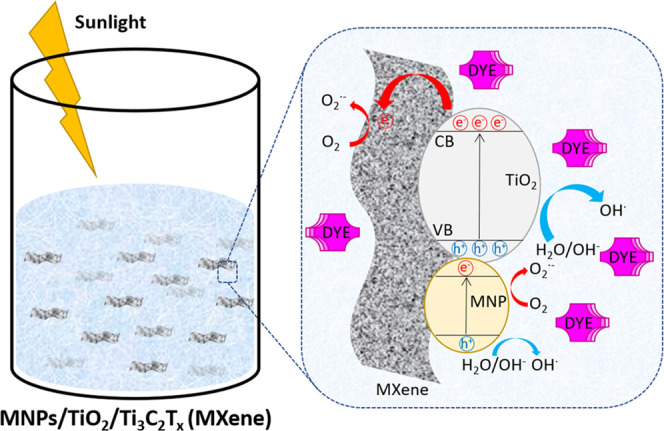
Schematic diagram illustrating the different potential mechanisms of the photoinduced charge-transfer process of Ti_3_C_2_T_*x*_ MXene modified with metal oxides (TiO_2_) and noble metal nanoparticles (MNP).

Based on the band structure alignments of the metal–semiconductor heterojunction and noble metal surface plasmon resonance charge injection effect, a possible photodegradation mechanism is depicted in Figure 12.

The typical photodegradation mechanism of organic molecules comprises redox reactions with the formation of free radicals, primarily generated by scavenging of photoinduced electrons by O_2_ molecules to form O_2_^•–^ anion radicals or by the oxidation of hydroxyl groups and water molecules, by photogenerated holes, to form OH^•^ radicals.^45,69^ The proposed degradation process, for the systems here reported, combines several mechanisms of charge transfer. One of the main mechanisms involves the movement of photogenerated electrons from the TiO_2_ conduction band (CB) to the valence band (VB). The photoinduced electrons can further migrate to Ti_3_C_2_T_*x*_ MXene sheets through the formed Schottky barrier at the TiO_2_/Ti_3_C_2_T_*x*_ interface, leaving the holes on the VB of TiO_2_. According to My Tran et al., this process promotes charge separation by reducing the chances of recombination and enhancing the lifetime of photogenerated electrons.^69^ In further detail, the photogenerated electrons would quickly migrate from TiO_2_ to the surface of MXene, which acts as an electron reservoir. The electron-rich reactive centers on the Ti_3_C_2_ surface are then responsible for O_2_^•–^ radical formation.^19,70,71^ On the other hand, the photogenerated holes can oxidize either the superficial hydroxyl groups on the TiO_2_ particles, the surrounding water molecules, and potentially the OH groups on the MXene surface, to form OH^•^ radicals.^69^

Decorating TiO_2_ nanostructures with transition-metal nanoparticles has been proven by several researchers to improve the photocatalytic properties of semiconductor nanoparticles, and our results further confirmed this trend.^2,19,20,36,71^ The presence of metal nanoparticles on the oxidized Ti_2_C MXene surface should provide additional reactive centers, especially toward dye adsorption, that could also result in the possibility of migration of photoinduced electrons to the surface of the metallic nanoparticles. According to the literature, the deposition of metal nanoparticles can significantly enhance the TiO_2_ photocatalytic activity. For example, Roşu et al. incorporated AgNP into graphene/TiO_2_ nanocomposites, which improved the catalyst light-absorption capacity and served as electron traps, reducing the likelihood of electron–hole recombination.^72^ This enhancement is generally attributed to the excitation of the SPR by visible-light radiation, confirming that metal NPs on TiO_2_ are effective and stable photocatalysts under solar radiation.^70,72^ When metal NPs absorb visible light, the surface electrons are excited to a higher energy state due to SPR effect; these electrons can further react with the oxygen molecules to form oxygen radicals (O_2_^•–^). Also, the holes photogenerated can accept electrons from the adsorbed photosensitized dye molecule; both mechanisms contribute to the degradation of the dye molecules.^73^ MNP/MXene heterojunctions are also characterized by a lower photoluminescence intensity when compared to their corresponding pristine substrates, generally associated with fast charge separation.^11^

It is therefore important to remark on the complexity of the processes and reactions that can coexist in MNP/TiO_2_/MXene systems that will end up having a synergic enhancement to the overall photodegradation of organic pollutants. Nevertheless, noble metals should be further studied through identifying the optimal metal content and metal geometry to reinforce their role in attaining high photocatalytic efficiency. Additionally, the experimental parameters, including catalyst loading, chemical nature of the dye, and irradiation wavelengths, play a significant role in promoting one mechanism over another.

## 3 Conclusions

This work has demonstrated a facile one-pot hydrothermal method to prepare oxidized MXene composites decorated with transition-metal nanoparticles (TiO_2_/Ti_3_C_2_T_*x*_, AgNPs/TiO_2_/Ti_3_C_2_T_*x*_, and PdNPs/TiO_2_/Ti_3_C_2_T_*x*_). The structure of the nanocomposites confirmed a stable formation of MNPs/TiO_2_/Ti_3_C_2_T_*x*_, and those TiO_2_ particles were nucleated on the surface of MXene. Additionally, the results indicate a superior performance for the AgNPs/TiO_2_/Ti_3_C_2_T_*x*_ photocatalyst, mainly ascribed to the positive role of anatase TiO_2_ and silver particles in enhancing light harvesting, dye adsorption, and charge separation. AgNPs/TiO_2_/Ti_3_C_2_T_*x*_ possessed higher degradation efficiencies toward MB and RhB under UV irradiation than most of the previously reported photocatalysts under similar experimental conditions, and its remarkable performance was confirmed by a 23% reduction of the total organic carbon after treating industrial wastewater. Owing to its facile fabrication and high photocatalytic performance, the AgNPs/TiO_2_/Ti_3_C_2_T_*x*_ composite has been proven a promising material for solar-light-based wastewater treatment.

## 4 Materials and Methods

### 4.1 Materials

MAX phase (Ti_3_AlC_2_) was purchased from Y-Carbon, Ltd., Ukraine. All of the other chemicals, hydrochloric acid, lithium fluoride, silver nitrate, and palladium(II) acetate, were purchased from Sigma-Aldrich, analytical grade, and were used as received. In all experiments, deionized (DI) reagent-grade water (≥18.2 MΩ) was used. The wastewater samples were collected from a local paper recycling factory.

### 4.2 Preparation of Delaminated (DL)-Ti_3_C_2_T*_x_* (MXene)

Multilayered (ML)-Ti_3_C_2_T_*x*_ MXene was prepared according to the procedure previously reported by Alhabeb et al.,^74^ with minor modifications. Briefly, 0.8 g of lithium fluoride was dissolved in 10 mL of 9 M hydrochloric acid in a Teflon container and was left under continuous stirring for 5 min to form in situ the HF etchant. Then, 0.5 g of the MAX phase (Ti_3_A1C_2_) was gradually added to the prepared etching solution, and the reaction was allowed to stir for 24 h at room temperature (RT). The resulting acidic black suspension was then quenched with DI water and washed by multiple centrifugation cycles (2000 RCF, 5 min) until the pH of the supernatant reached ≥5. Delamination of the ML MXene was carried out by probe sonication (on–off pulsing of 2 s, frequency of 20 kHz, and power of 120 W) for 1 h. Finally, the colloidal solution of MXene was further centrifuged (1500 RCF, 30 min) and freeze-dried to obtain the desired single- or few-layered DL Ti_3_C_2_T_*x*_ MXene powder.

### 4.3 Preparation of Metal NPs/TiO_2_/Ti_3_C_2_T_*x*_ Composites

AgNPs/TiO_2_/Ti_3_C_2_T_*x*_ composites were prepared by hydrothermal treatment of an aqueous solution containing ML Ti_3_C_2_T_*x*_ nanosheets and AgNO_3_ salt. In detail, 50 mg of ML MXene was dissolved into 50 mL of deionized (DI) water, and it was delaminated in situ by stirring and probe sonication for 5 and 10 min, respectively, at RT to form a homogeneous colloidal solution of DL Ti_3_C_2_T_*x*_. Then, 19.7 mg of silver nitrate (corresponding to 12.5 mg of Ag) was added into the prepared colloidal solution, followed by stirring and probe sonication for 5 and 10 min, respectively. Subsequently, the solution was transferred into a 100 mL Teflon-lined stainless steel container. The hydrothermal reaction was conducted at 160 °C for 12 h with the heat ramp-up of 2 °C/min. Then, the Teflon container was allowed to cool to room temperature. The product was collected by centrifugation (2000 RCF, 5 min), washed, and then freeze-dried for 48 h to obtain the composite powder.

Similarly, PdNPs/TiO_2_/Ti_3_C_2_T_*x*_ was prepared via adding 26.4 mg of palladium(II) acetate (corresponding to 12.5 mg of Pd) into the prepared colloidal solution of MXene before conducting hydrothermal treatment. For the preparation of TiO_2_/Ti_3_C_2_T_*x*_, no metal salts were added to the colloidal solution of MXene prior to the hydrothermal treatment.

### 4.4 Characterization

The crystal structure of the samples was determined by a Bruker D8 Advance X-ray diffractometer (XRD) using Cu Kα radiation (λ = 1.5418 Å) at instrument settings of 40 kV and 40 mA. The elemental and functional group compositions of the samples were analyzed by Thermo Fisher Scientific ESCALAB 250Xi X-ray photoelectron spectroscopy (XPS), using Al Kα excitation radiation (25 W, *h*ν = 1486.5 eV) and 1 eV energy resolution. Composite morphology was analyzed with a FEI Talos F200X transmission electron microscope (TEM) operating at an accelerating voltage of 200 kV, whereas the localized elemental mapping was screened by high-angle annular dark-field scanning transmission electron microscopy (HAADF-STEM). FEI model QuantaFEG 650 scanning electron microscopy (SEM) with 5 kV acceleration voltage and Bruker EDS energy-dispersive X-ray spectroscopy were also used for morphology study and element visualization. The ultraviolet–visible (UV–vis) absorbance spectra were collected using a Jasco V-670 UV–visible spectrophotometer. The photoluminescence (PL) spectra were investigated by a PL spectrometer (Horiba, iHR 320, MicOS) with an excitation wavelength of 325 nm. The concentration of organic carbon in the wastewater was analyzed using a combustion-type total organic carbon analyzer (Shimadzu, model TOC-L, Japan).

### 4.5 Photocatalytic Degradation of Dyes

**Figure 13:**
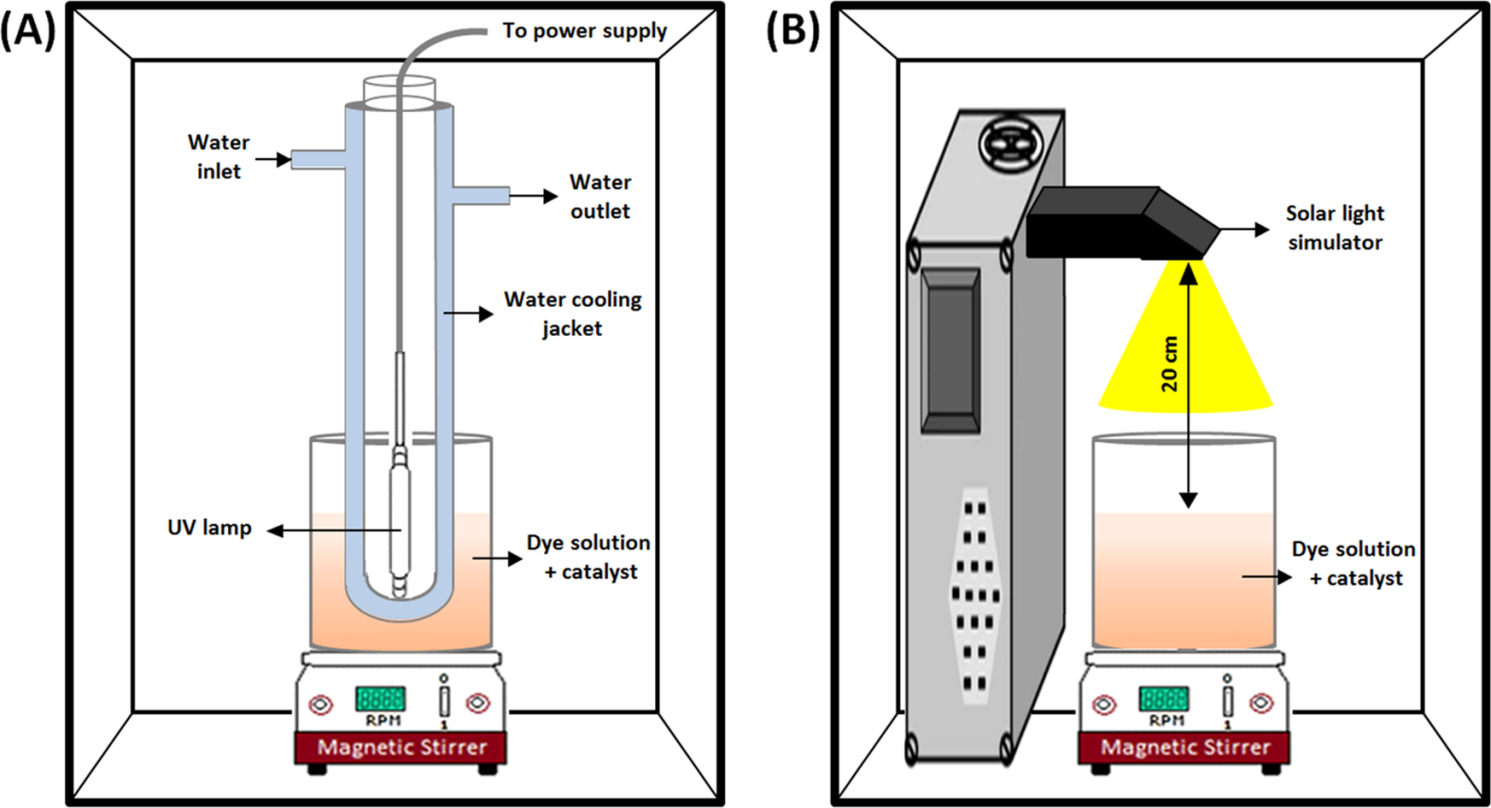
Schematic diagram of the photocatalytic degradation setup, (A) under UV light and (B) under the solar simulator.

The photocatalytic degradation performance of the synthesized photocatalysts was evaluated under UV light irradiation against rhodamine B (RhB, 10 mg/L) and methylene blue (MB, 10 mg/L). Standard amounts of the photocatalysts (1, 2.5, 5, and 10 mg) were dispersed in 100 mL of the prepared organic dye solutions. Thus, different experiments with photocatalyst-to-dye-pollutant ratios (1:1, 2.5:1, 5:1, and 10:1) were conducted. A UV protection cabinet equipped with a UV medium-pressure immersion lamp (model TQ 150; no. 5600 1725; brand Heraeus Noblelight) was used during the experiments (Figure 13A). The dye/photocatalyst mixture was homogenized with a magnetic stirrer. Prior to irradiation, the dye/photocatalyst mixture was stirred for 30 min to establish an adsorption–desorption equilibrium. The UV light source employed in this study is a 400 W lamp with a line spectrum in the ultraviolet and visible range (200–600 nm), with a high power output density of about 100 W/cm^2^ in the UVC range (200–300 nm). After the light was turned on, aliquots of 1 mL were taken at fixed time intervals and were centrifuged to separate the photocatalysts. Each photocatalytic degradation test was done in duplicate, and the average values were presented. MB and RhB concentrations were measured by a UV–vis spectrometer, and the removal efficiency (%) was calculated by the following equation (eq 2)
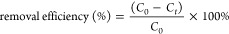
2where *C*_0_ and *C_t_* (mg/L) are the initial and final concentrations of MB or RhB at time *t*, respectively.

For the solar light photocatalytic degradation experiment (Figure 13B), various photocatalyst-to-organic-pollutant ratios (5:1, 10:1, 20:1, and 30:1) were studied. The photocatalyst (5, 10, 20, or 30 mg) was dispersed in 100 mL of MB (10 mg/L) or RhB (10 mg/L) dye solutions in a 200 mL beaker reactor. This was placed 20 cm away from a solar light simulator (IEC/JIS/ASTM, 450 W xenon, 100 mW/cm^2^). The procedures for mixture stirring, sample extraction, and sample analysis were done as previously described for experiments under UV light. The schematic representations of both experimental setups are shown in Figure 13.

Tap water and wastewater samples, collected from a local wastepaper recycling factory (total carbon 2401 mg/L; total organic carbon 2355 mg/L), were used to assess the practical application of the most performing photocatalyst. The wastewater was filtered by Whatman filter paper 41 (20–25 μm pore size) to remove suspended pulp material that may cause light shielding/scattering. Moreover, for a better comparison with the other photocatalytic degradation experiments conducted in this study, the wastewater was diluted with DI water to adjust its total organic carbon content to about 10 mg/L. Both tap and wastewater samples were also spiked with MB dye to obtain a final MB concentration of 10 mg/L. Syringe filters (0.45 μm poly(vinylidene difluoride) (PVDF), Millipore) were used while carrying out total organic carbon analysis for both tap water and wastewater samples.

## Supporting Information Available

SEM, TEM, and HAADF-STEM images; XPS spectra, UV–vis absorption spectrum, PL spectra, light intensity spectrum of solar light; kinetic trends and photodegradation performance of coexisting MB and RhB by AgNPs/TiO_2_/Ti_3_C_2_T_*x*_ (PDF)
