# Supplementary material for: Efficient Photocatalytic Degradation of Organic Dyes by AgNPs/TiO2/Ti3C2Tx MXene Composites under UV and Solar Light
Source: ACS Omega. 2021 Dec 1;6(49):33325–38. doi: 10.1021/acsomega.1c03189 (PMC8674905; doi:10.1021/acsomega.1c03189)
Supplement: Supplementary file 1 — ao1c03189_si_001.pdf [file ao1c03189_si_001.pdf]

Supporting Information

Efficient Photocatalytic Degradation of Organic Dyes by AgNPs/TiO_2/$Ti_3C_2T_x$ MXene Composite Under UV and Solar Light

*Zakarya Othman[a,b], Alessandro Sinopoli[a], Hamish R. Mackey[b], Khaled A. Mahmoud[a]**

[a]Qatar Environment and Energy Research Institute (QEERI), Hamad Bin Khalifa University, Qatar Foundation, P.O. Box 34110, Doha, Qatar

[b]Division of Sustainable Development, College of Science and Engineering, Hamad bin Khalifa University, Qatar Foundation, P.O. Box 34110, Doha, Qatar

[*] Corresponding author**.** E-mail: kmahmoud@hbku.edu.qa, Phone: +974 44541694

Table S1: XPS results of the elemental composition

Elements	Element %			
	$Ti_3C_2T_x$	$TiO_2/Ti_3C_2T_x$	$AgNPs/TiO_2/Ti_3C_2T_x$	$PdNPs/TiO_2/Ti_3C_2T_x$
C 1s	26.04	21.86	22.76	18.79
Ti 2p	17.14	28.12	24.58	20.04
O 1s	18.45	39.77	43.36	48.35
Ag 3d	0.00	0.00	1.69	0.00
Pd 3d	0.00	0.00	0.00	4.00
F 1s	38.37	10.25	7.61	8.82

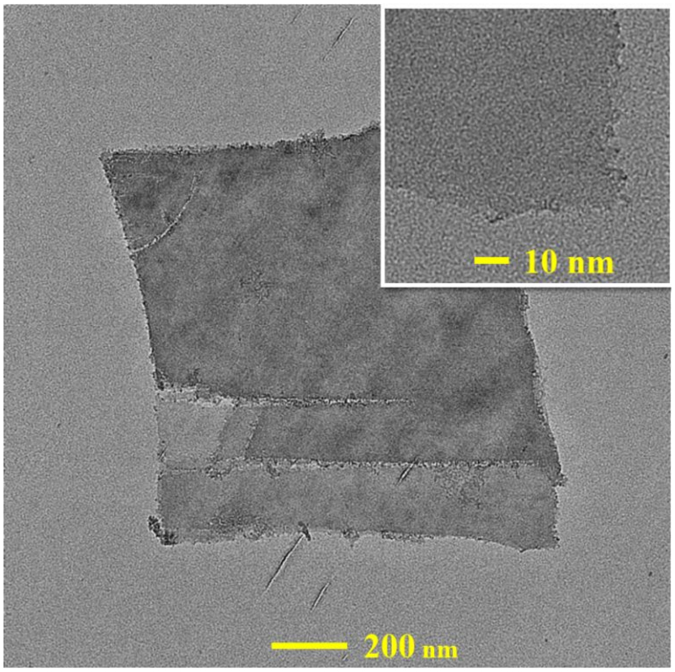

Figure S1: TEM images of $Ti_3C_2T_x$ MXene sheets

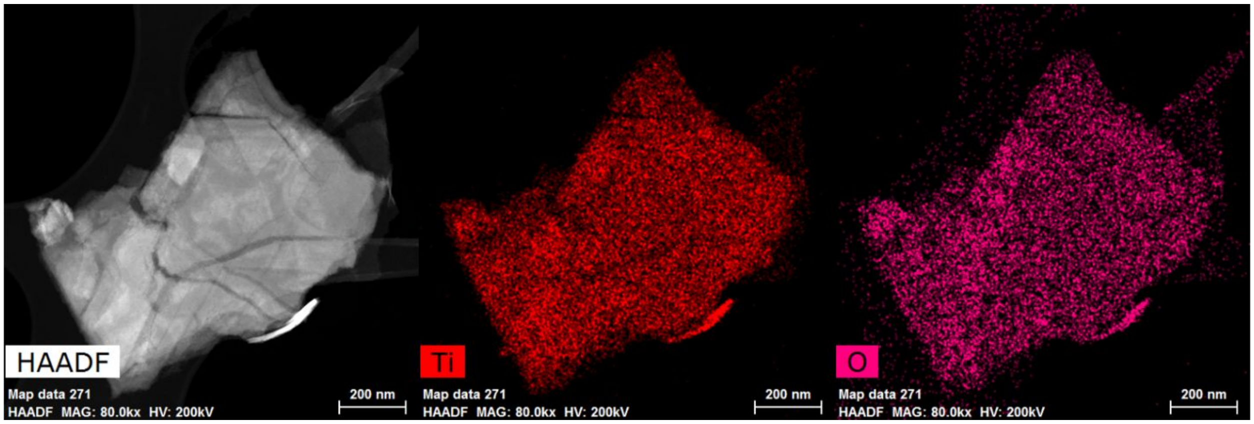

Figure S2: Element mapping of $Ti_3C_2T_x$ by high angle annular dark field scanning transmission electron microscopy (HAADF-STEM)

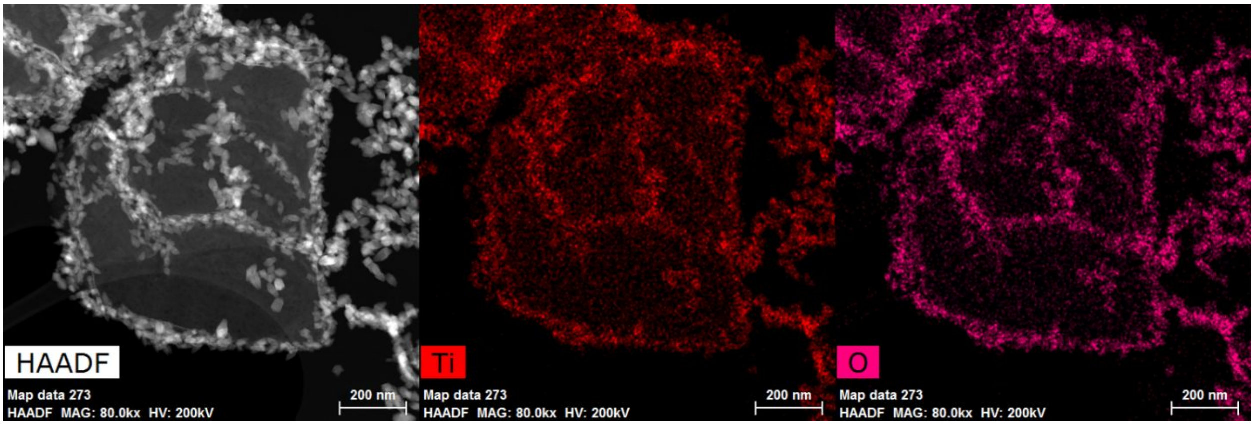

Figure S3: Element mapping of $TiO_2/Ti_3C_2T_x$ by high angle annular dark field scanning transmission electron microscopy (HAADF-STEM)

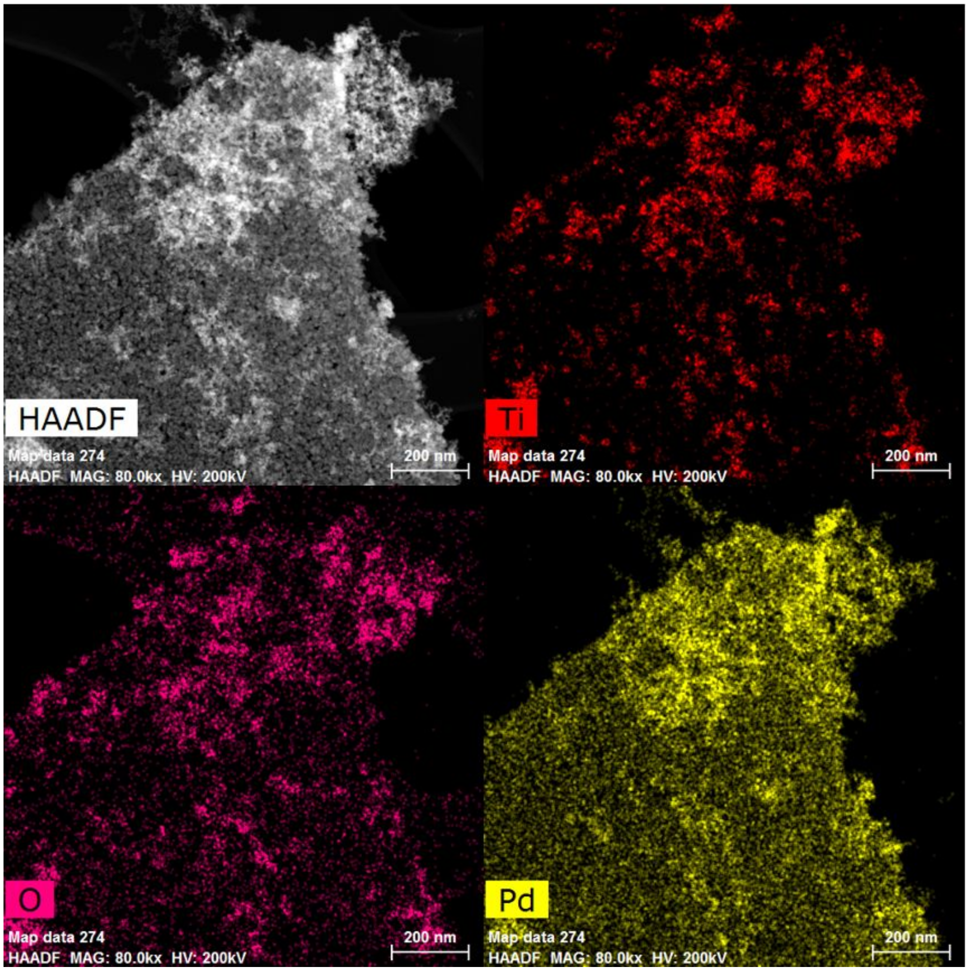

Figure S4: Element mapping of PdNPs/TiO_2/$Ti_3C_2T_x$,by high angle annular dark field scanning transmission electron microscopy (HAADF-STEM)

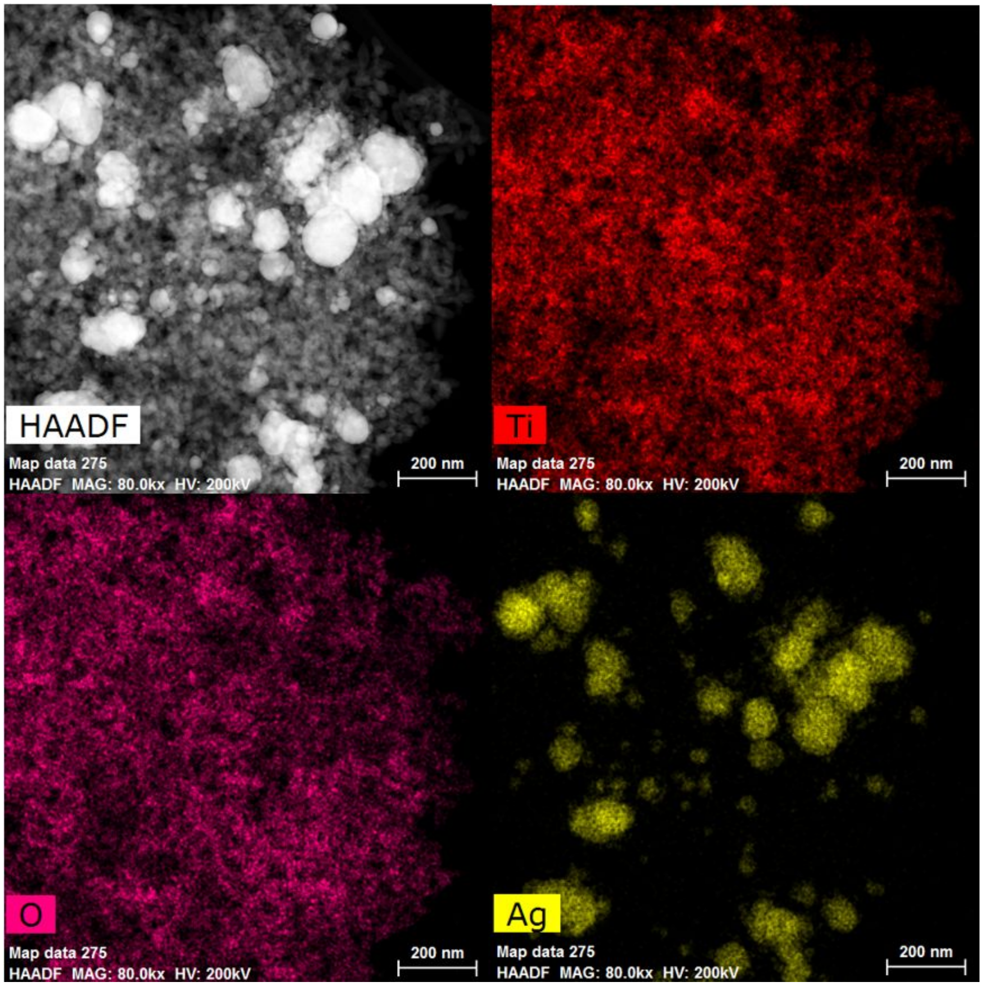

Figure S5: Element mapping of AgNPs/TiO_2/$Ti_3C_2T_x$,by high angle annular dark field scanning transmission electron microscopy (HAADF-STEM)

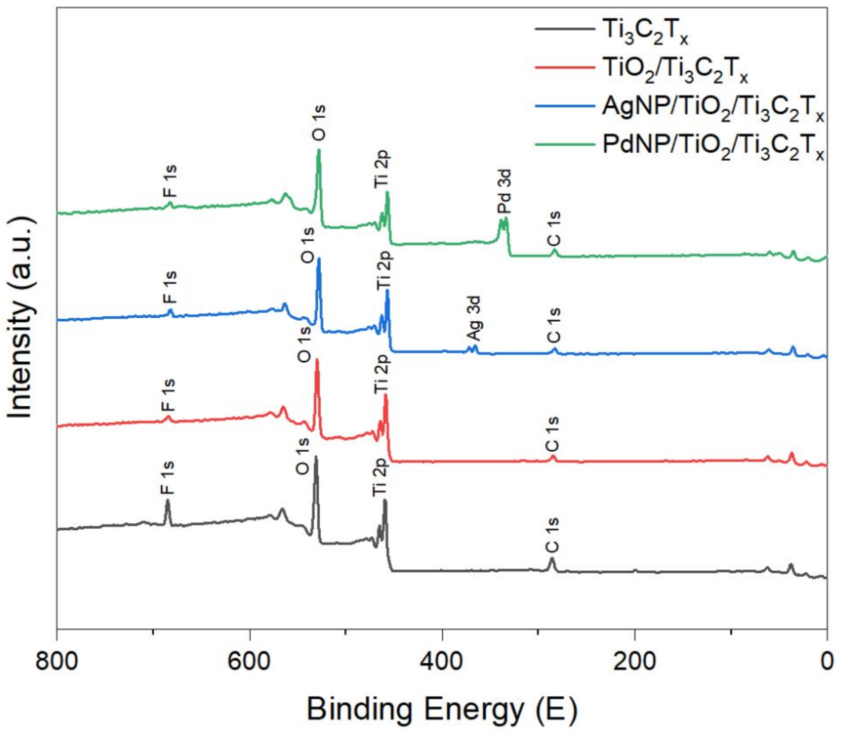

Figure S6: XPS spectrum for $Ti_3C_2T_x$, $TiO_2/Ti_3C_2T_x$, $AgNPs/TiO_2/Ti_3C_2T_x$ and $PdNPs/TiO_2/Ti_3C_2T_x$, XPS

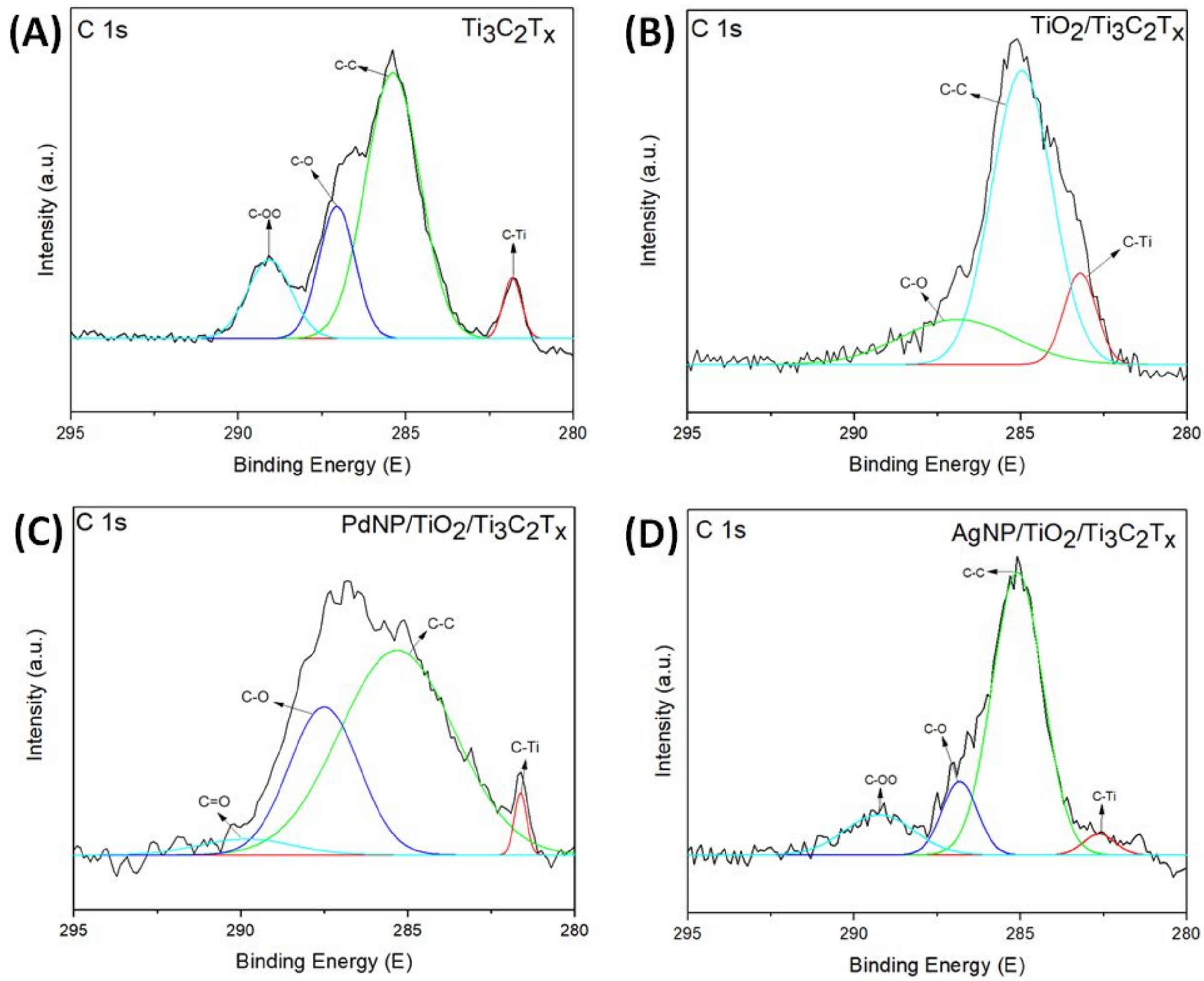

Figure S7: C 1s XPS spectrums for (A) $Ti_3C_2T_x$, (B)$TiO_2/Ti_3C_2T_x$, (C) PdNPs/$TiO_2/Ti_3C_2T_x$, and (D) AgNPs/$TiO_2/Ti_3C_2T_x$ photocatalysts.

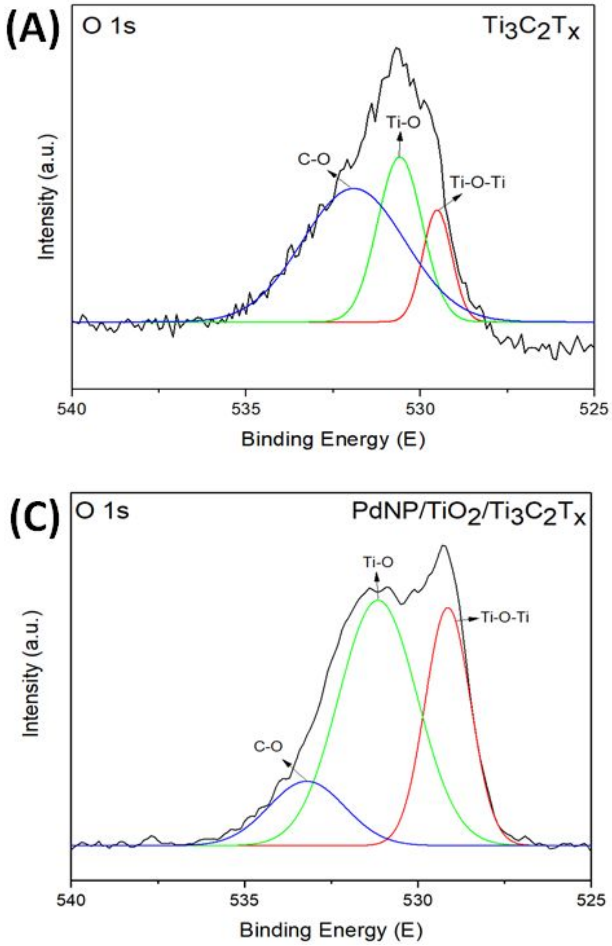

Figure S8: O 1s XPS spectrums for (A) $Ti_3C_2T_x$, (B)$TiO_2/Ti_3C_2T_x$, (C) PdNPs/$TiO_2/Ti_3C_2T_x$, and (D) AgNPs/$TiO_2/Ti_3C_2T_x$ photocatalysts.

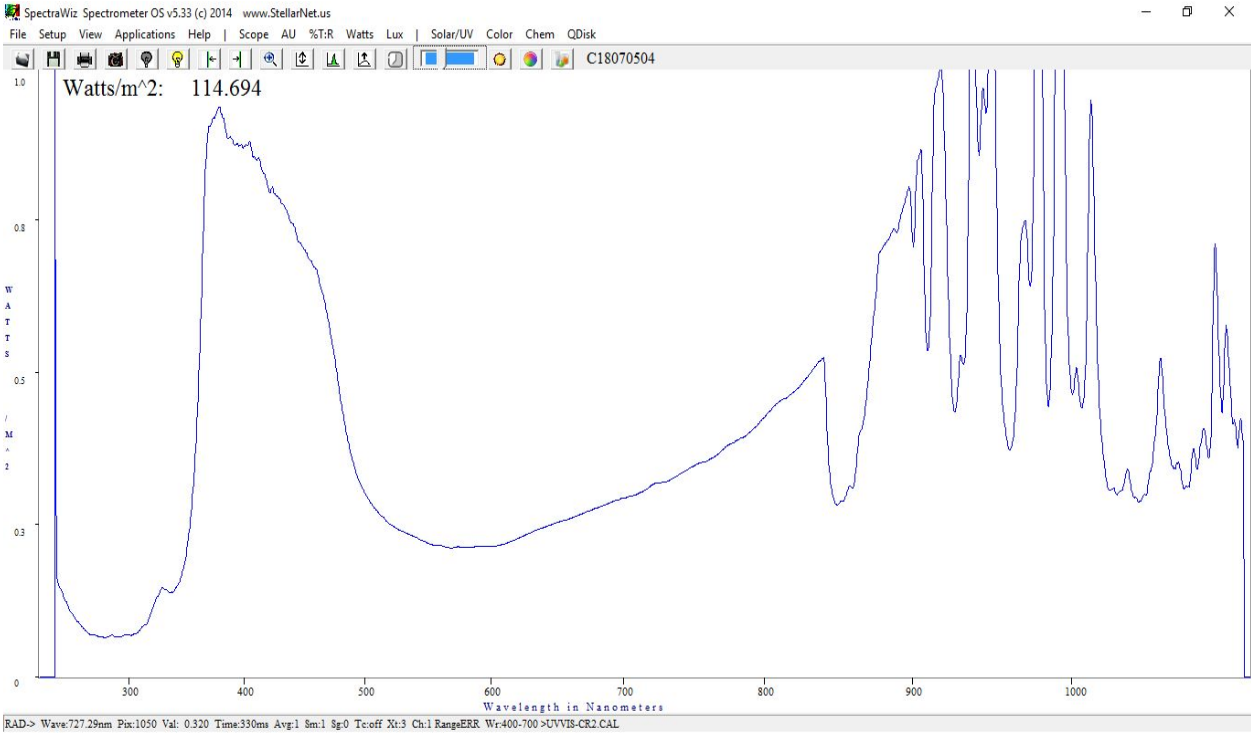

Figure S9: Light intensity spectrum of solar light simulator as measured by StellarNet Miniature UV-VIS BlueWave spectrometer model

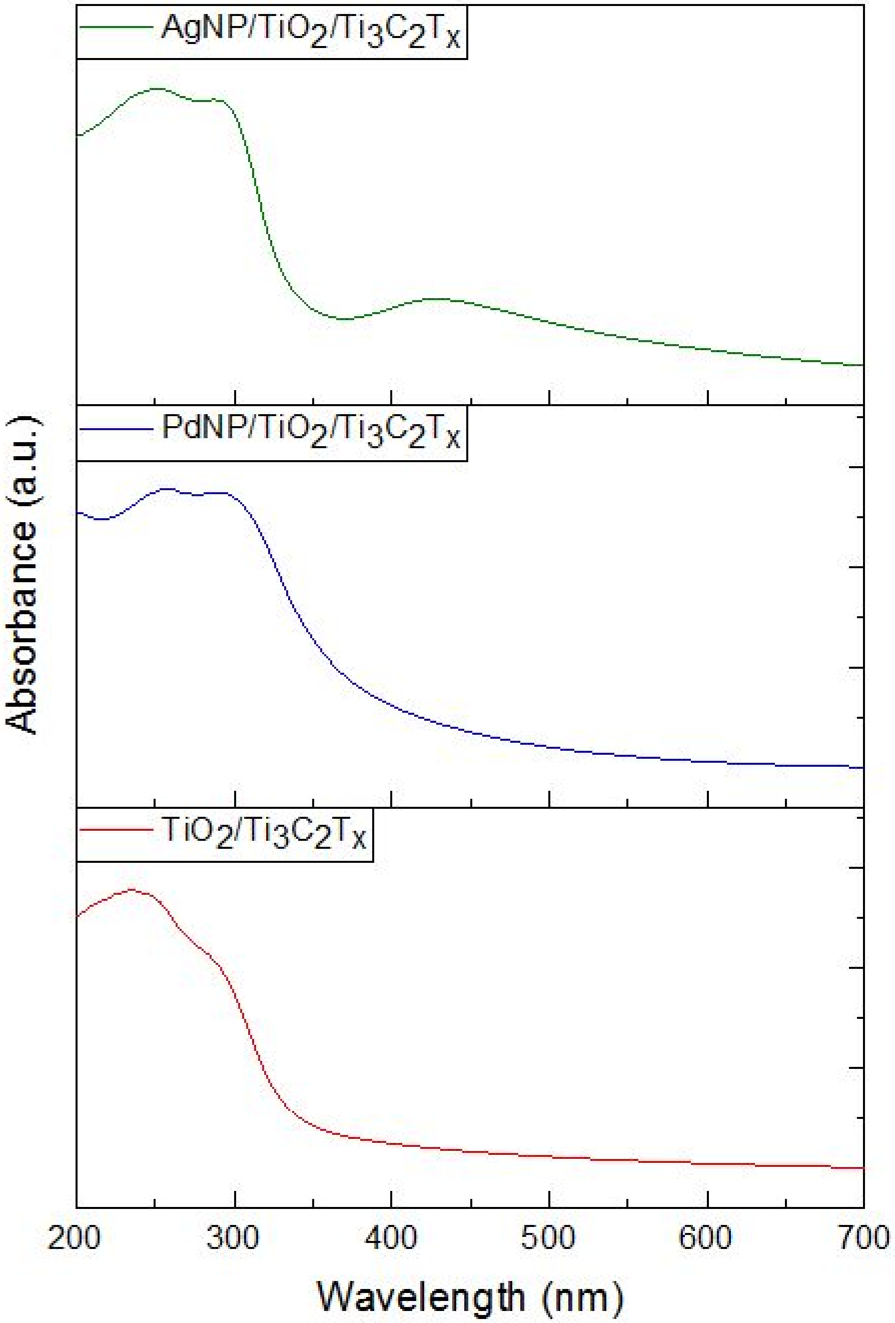

Figure S10: UV-vis absorption spectrum of $TiO_2/Ti_3C_2T_x$, AgNPs/$TiO_2/Ti_3C_2T_x$, and PdNPs/$TiO_2/Ti_3C_2T_x$ composites

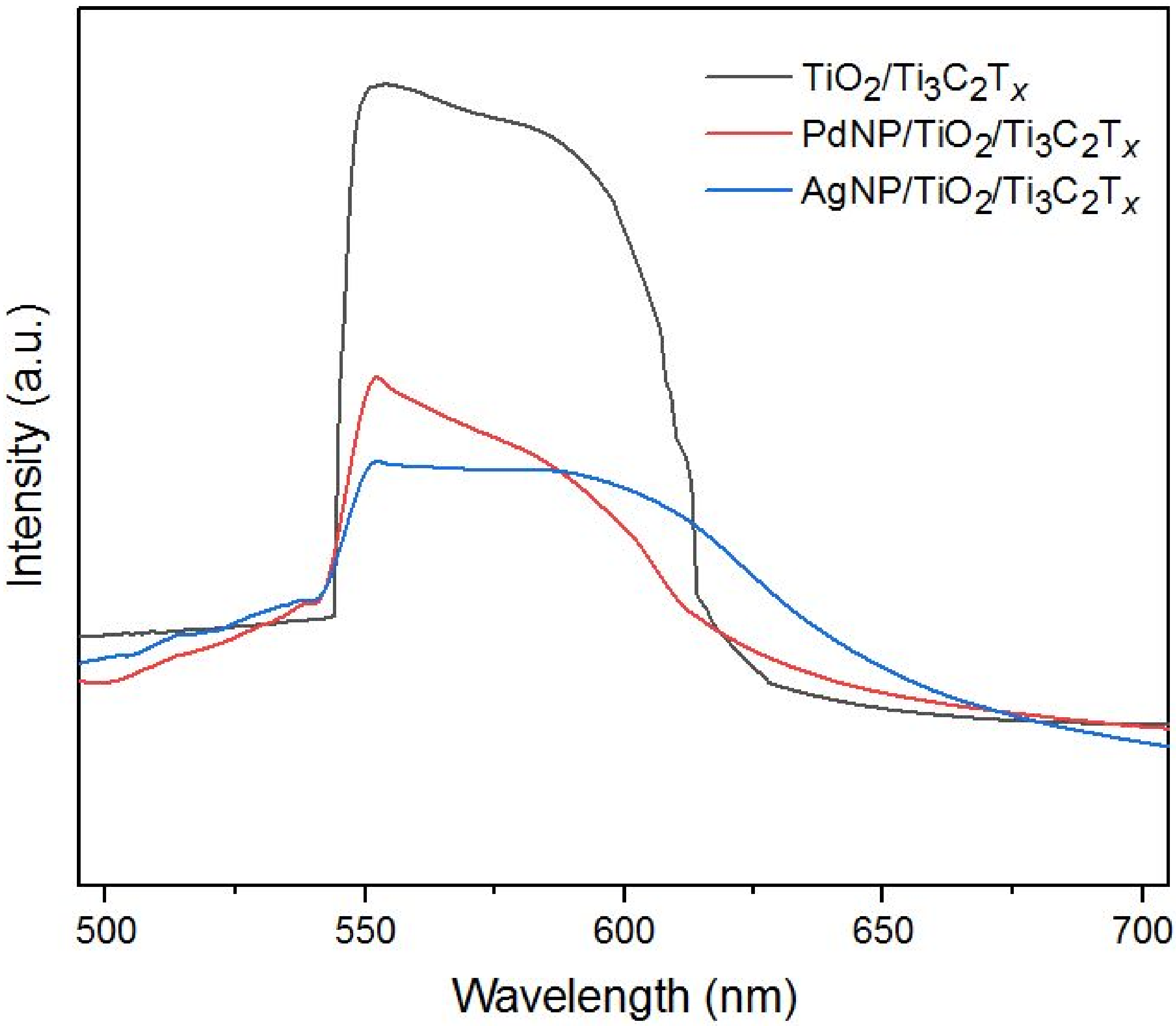

Figure S11: PL spectra of $TiO_2/Ti_3C_2T_x$, AgNPs/$TiO_2/Ti_3C_2T_x$, and PdNPs/$TiO_2/Ti_3C_2T_x$ composites

Pseudo-first order kinetic model

Photocatalysis kinetics generally follow Langmuir−Hinshelwood (LH) kinetic model [1].

$$-\frac{dC}{dt} = \frac{kKC}{1+KC} \qquad (1)$$

where k (mg/L/min) is the rate constant involving various parameters, such as mass of photocatalyst and light intensity, K (L/mg) is the adsorption constant at the catalyst surface, C(mg/L) is the dye concentration and t is the time (min). At low initial concentrations of the dye compared to photogenerated radicals, the term KC may be disregarded with respect to 1 and the rate (eq 1) may be approximated to a simpler pseudo-first order [2]

$$-\frac{dC}{dt} = kKC = k_{app}C \qquad (2)$$

where k_{app} is the apparent rate constant (min^{-1}) of the resulting pseudo first-order relationship, which after integration is

$$ln\frac{C_t}{C_0} = -k_{app}\,t \quad or \quad ln\frac{C_0}{C_t} = k_{app}\,t \qquad (3)$$

where, C_o and C_t are the initial and final concentration of MB or RhB, respectively.

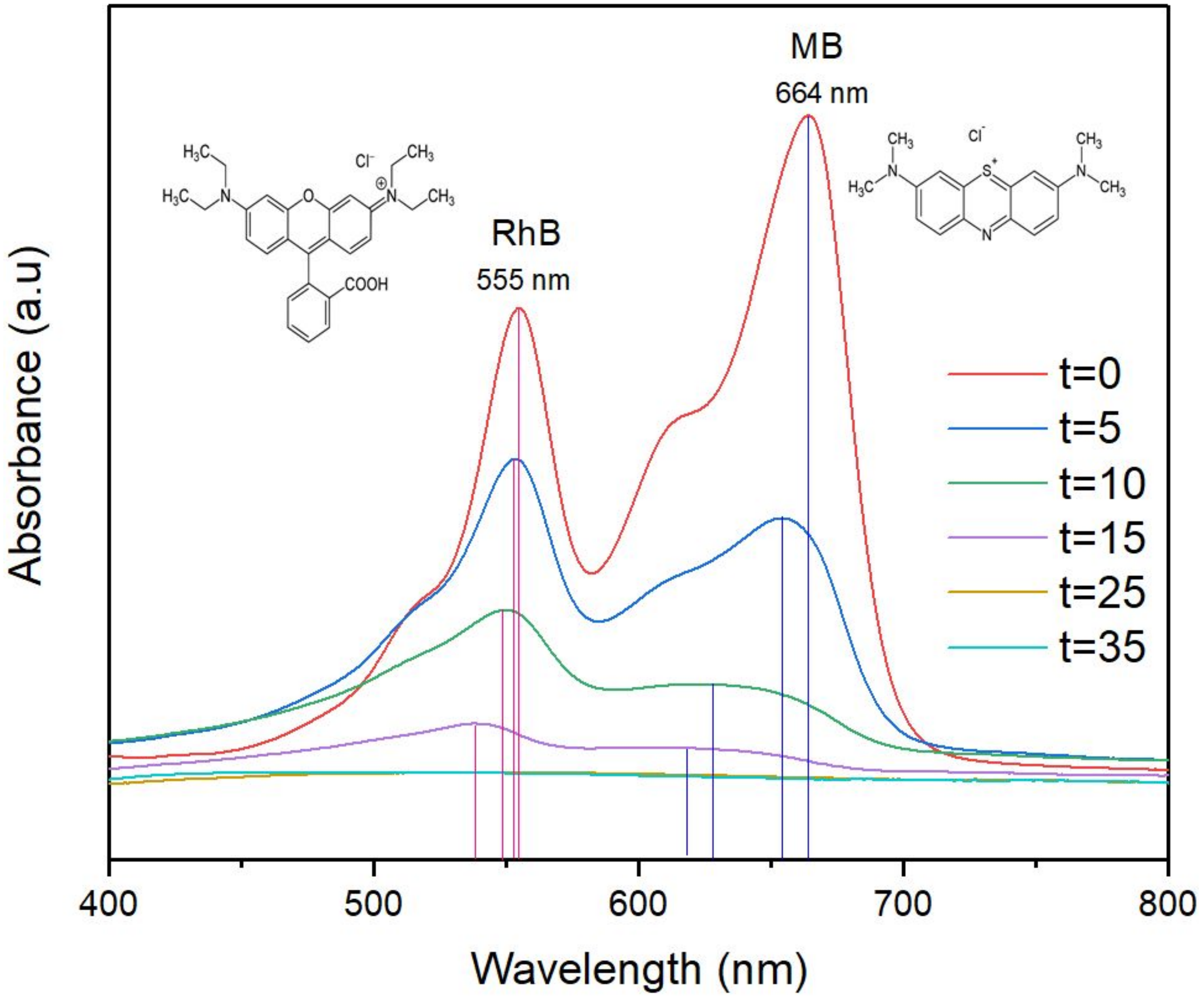

Figure S12: quenching of the UV−vis spectrum during degradation kinetic with AgNPs/TiO_2/$Ti_3C_2T_x$ against both MB and RhB under UV irradiation

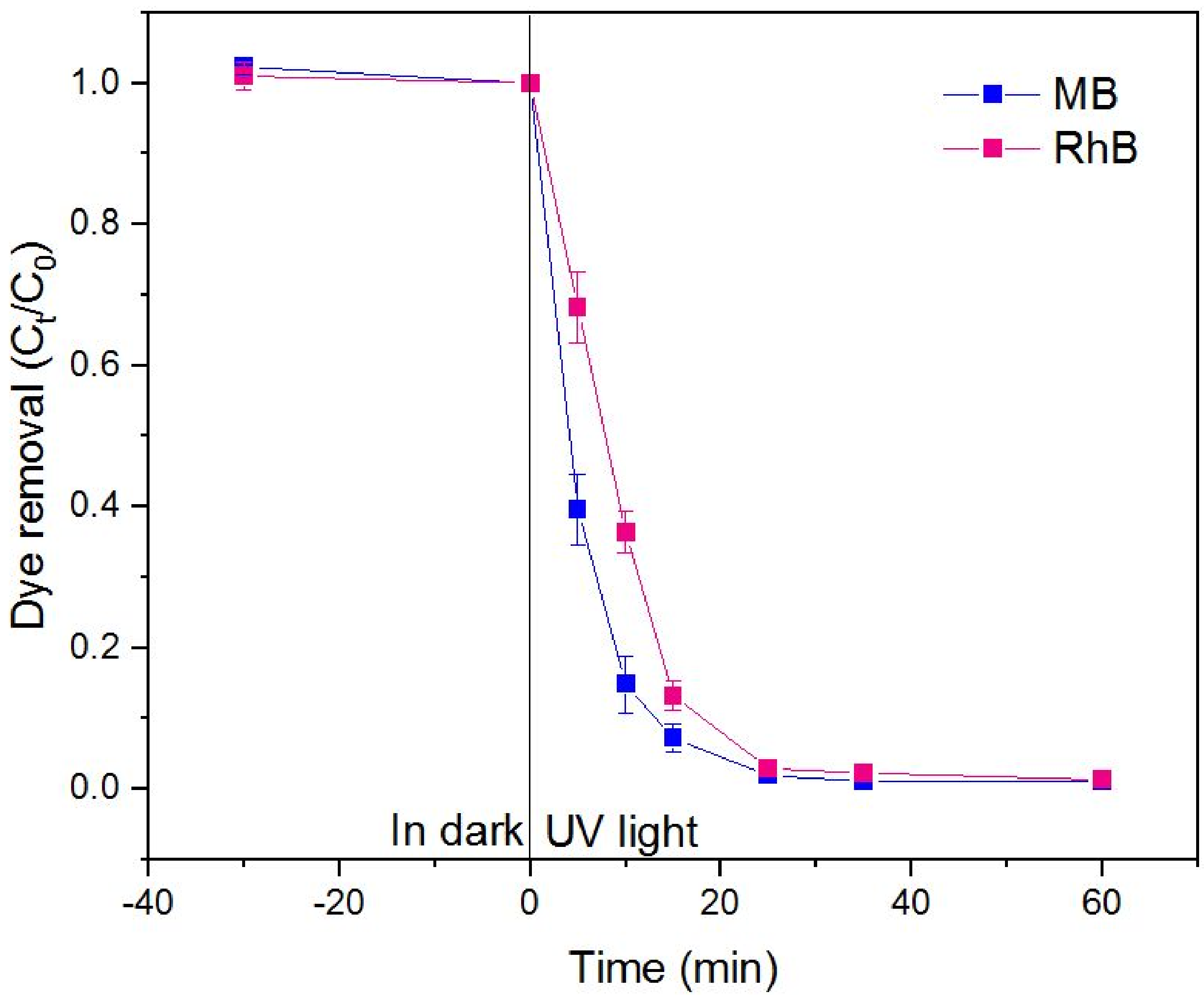

Figure S13: Effect of UV irradiation time on photodegradation of coexisting MB and RhB by AgNPs/TiO_2/$Ti_3C_2T_x$ (2.5:1 photocatalysts to dye ratio)

References:

1. Friedmann, D.; Mendive, C.; Bahnemann, D., TiO2 for water treatment: Parameters affecting the kinetics and mechanisms of photocatalysis. *Appl. Catal., B* **2010,** *99* (3), 398-406.
2. Petrella, A.; Boghetich, G.; Petrella, M.; Mastrorilli, P.; Petruzzelli, V.; Petruzzelli, D., Photocatalytic Degradation of Azo Dyes. Pilot Plant Investigation. *Ind. Eng. Chem. Res.* **2014,** *53* (7), 2566-2571.
